# LncRNA CAIF inhibits autophagy and attenuates myocardial infarction by blocking p53-mediated myocardin transcription

**DOI:** 10.1038/s41467-017-02280-y

**Published:** 2018-01-02

**Authors:** Cui-Yun Liu, Yu-Hui Zhang, Rui-Bei Li, Lu-Yu Zhou, Tao An, Rong-Cheng Zhang, Mei Zhai, Yan Huang, Kao-Wen Yan, Yan-Han Dong, Murugavel Ponnusamy, Chan Shan, Sheng Xu, Qi Wang, Yan-Hui Zhang, Jian Zhang, Kun Wang

**Affiliations:** 1 Center for Developmental Cardiology, Institute for Translational Medicine, College of Medicine, Qingdao University, Qingdao, 266021 China; 2 State Key Laboratory of Cardiovascular Disease, Heart Failure center, Fuwai Hospital, National Center for Cardiovascular Diseases, Chinese Academy of Medical Sciences, Peking Union Medical College, Beijing, 100037 China; 3 School of Professional Studies, Northwestern University, Chicago, IL 60611 USA

## Abstract

Increasing evidence suggests that long noncoding RNAs (lncRNAs) play crucial roles in various biological processes. However, little is known about the effects of lncRNAs on autophagy. Here we report that a lncRNA, termed cardiac autophagy inhibitory factor (CAIF), suppresses cardiac autophagy and attenuates myocardial infarction by targeting p53-mediated myocardin transcription. Myocardin expression is upregulated upon H_2_O_2_ and ischemia/reperfusion, and knockdown of myocardin inhibits autophagy and attenuates myocardial infarction. p53 regulates cardiomyocytes autophagy and myocardial ischemia/reperfusion injury by regulating myocardin expression. CAIF directly binds to p53 protein and blocks p53-mediated myocardin transcription, which results in the decrease of myocardin expression. Collectively, our data reveal a novel CAIF-p53-myocardin axis as a critical regulator in cardiomyocyte autophagy, which will be potential therapeutic targets in treatment of defective autophagy-associated cardiovascular diseases.

## Abstract (web summary)

Little is known about the role of long lncRNAs in autophagy. The authors identify lncCAIF, and show that it suppresses cardiac autophagy and attenuates myocardial infarction by targeting p53 -mediated transcription of myocardin.

## Introduction

Autophagy is an evolutionarily conserved intracellular degradation process, which maintains cellular homeostasis by removing damaged proteins and organelle turnover. Autophagy has been demonstrated to play a wide variety of physiological and pathophysiological roles in many kinds of cells and tissues. Autophagy dysregulation is associated with a number of cardiac diseases, including dilated cardiomyopathy, ischemic heart disease, and heart failure^1–3^. Although some studies suggest that autophagic cell death plays a pivotal role in cardiac disease, hitherto, there is no effective treatment for the autophagy-related heart disease and heart failure. Thus, it is of great importance to explore and reveal the molecular mechanism underlying the regulation of autophagy. Understanding the mechanism of autophagy will provide a novel interventional strategy for treating cardiovascular diseases and heart failure.

LncRNAs are important class of noncoding RNA that are characterized by their length longer than 200 nt. LncRNAs have been identified in many types of cells and tissues. LncRNAs play vital role in various normal physiological and pathological conditions including cell differentiation, metabolism and cancer progression^4–6^. LncRNAs regulate molecules by multiple mechanisms, including epigenetic regulation, genomic imprinting, RNA stability, RNA alternative splice and microRNA regulation^7,8^. Emerging evidences suggest that lncRNAs are involved in the regulation of cardiac diseases^9,10^. However, the influence of lncRNAs in the regulation of autophagy in the heart remains largely unknown.

P53 is a tumor suppressor protein and is widely known for its role as a transcription factor. It is well known that p53 activates genes that regulate cell cycle checkpoints, DNA damage and repair, and apoptosis^11^. Studies show that there is an important relationship between p53 and autophagy. Deletion or inhibition of p53 affects autophagy in several cell lines, including human, mouse and nematode cells^12^. However, the autophagy regulatory function of p53 in cardiomyocytes remains largely unknown.

Myocardin is a nuclear protein and a transcriptional coactivator of serum response factor that is specifically expressed in the smooth muscle and cardiac muscle^13^. Myocardin dysfunction in cardiomyocytes triggers apoptosis^14^. Myocardin is important for the maintenance of heart function. Myocardin transactivates the Bmp10 and regulates cardiomyocyte proliferation and apoptosis in the embryonic heart^15^. Although myocardin is of great importance to some pathology and physiology process in the heart, it is not yet clear whether myocardin can regulate autophagic program in cardiomyocyte. Our present study reveals that myocardin positively modulates the autophagic process in cardiomyocytes and it promotes myocardial infarction. The knockdown of myocardin inhibits autophagic cell death and attenuates ischemic injury induced increase of myocardial infarction size. We demonstrate that p53 activates myocardin transcription. It upregulates autophagy and myocardial infarction by increasing myocardin expression. Further, we observed that a lncRNA, termed cardiac autophagy inhibitory factor (CAIF), directly binds to p53 protein and blocks its binding to the promoter region of myocardin. Our study indicates that CAIF is able to inhibit cell death and attenuate myocardial infarction in the heart through targeting the p53/myocardin dependent autophagy pathway. Our findings offer a better understanding about the interaction between lncRNA and proteins involved in the regulation of autophagy. Collectively, this study provides a novel evidence that lncRNA modulates cardiomyocyte autophagy and protects the cardiac tissue from myocardial infarction.

## Results

### Myocardin mediates autophagy and cell death in cardiomyocyte

**Fig. 1:**
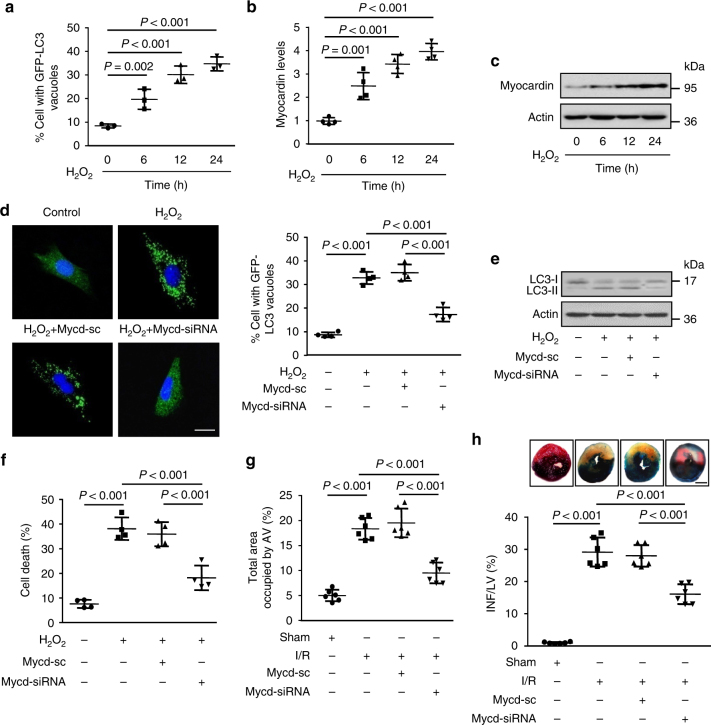
Myocardin participates in the regulation of autophagy. **a** H_2_O_2_ induces autophagosome formation. Cardiomyocytes were infected with GFP-LC3 and then treated with H_2_O_2_ at the indicated time. The extent of autophagy was assessed by analyzing staining patterns of GFP-LC3, and quantitation of autophagy was shown. *n* = 3. **b**, **c** H_2_O_2_ induces an increase in myocardin levels. Cardiomyocytes were treated with H_2_O_2_ at indicated time. Myocardin mRNA levels (**b**) and protein levels (**c**) were analyzed. *n* = 4. **d** Knockdown of myocardin inhibits punctate accumulations of GFP-LC3 induced by H_2_O_2_. Cardiomyocytes were infected with adenoviral myocardin siRNA (Mycd-siRNA) or its scramble form (Mycd-sc), then infected with GFP-LC3. Twenty-four hours after infection cells were treated with H_2_O_2_. Representative photos of GFP-LC3 cells were shown in the left panel (Bar = 20 μm) and the percentage of cells with GFP-LC3 puncta was quantified in the right panel. *n* = 4. **e** Representative immunoblot for conversion of LC3-I to LC3-II. Cardiomyocytes were infected with adenoviral Mycd-siRNA or Mycd-sc. Twenty-four hours after infection cells were treated with H_2_O_2_. The positions of LC3-I and LC3-II are indicated. *n* = 3. **f** Knockdown of myocardin reduces cell death. Cells were treated as described in (**e**). Quantitation of cell death was shown. *n* = 4. **g** Myocardin knockdown suppresses autophagy in vivo. Mice were injected with Mycd-siRNA or Mycd-sc as described in methods section, and then were subjected to 45 min ischemia and 3 h reperfusion (I/R). Quantification of autophagic vacuoles in the area-at risk was shown. *n* = 6. **h** Knockdown of myocardin reduces myocardial infarction upon I/R in vivo. Mice were treated as described in (**g**). The upper panels are representative photos of midventricular myocardial slices (Bar = 2 mm). The low panel shows infarct sizes. LV left ventricle, INF infarct area. *n* = 6 mice

Oxidative stress play critical role in the development of cardiovascular problems and cardiomyocytes are more prone to oxidant injury. It is well known that oxidants such as hydrogen peroxide (H_2_O_2_) can induces autophagy process in cardiomyocytes and the dysregulation of autophagy causes cardiomyocyte cell death. To test whether myocardin participates in the regulation of autophagy in cardiomyocytes, we treated cardiomyocytes with H_2_O_2_ to induce autophagy and examined the expression pattern of myocardin. In cardiomyocytes, H_2_O_2_ exposure increased the autophagy as indicated by accumulation of GFP-LC3 punctuate structures (Fig. 1a), which was accompanied by a time dependent increase of myocardin expression (Fig. 1b, c). These results indicate that the level of myocardin is increased during H_2_O_2_-induced autophagy in cardiomyocytes. Next, we silenced the expression of myocardin and investigated its impact on H_2_O_2_ induced autophagy. The knockdown of myocardin (Supplementary Fig. 1a) remarkably reduced H_2_O_2_-induced autophagic process as shown by a significant decrease in punctuate accumulations of GFP-LC3 (Fig. 1d and Supplementary Fig. 1b). In addition, H_2_O_2_ induced increased of LC3-II expression was significantly decreased by silencing of myocardin with siRNA (Fig. 1e). Autophagic flux denotes the dynamic process of autophagy, which is a reliable indicator of autophagic activity. We therefore measured the autophagic flux using tandem mRFP-GFP-LC3 fluorescence analysis^16–18^ in cardiomyocytes. The yellow puncta, which are the combination of GFP and RFP fluorescence, indicate autophagosomes. The red puncta indicate autolysosomes, where green fluorescences of GFP-LC3 puncta are quenched due to the acidic environment. H_2_O_2_ induced autophagic flux in cardiomyocytes, as evidenced by the increased number of RFP-LC3 autolysosomes, whereas the knockdown of myocardin efficiently attenuated the H_2_O_2_-induced increase in the autophagic flux (Supplementary Fig. 1c). The autophagic cell death is recognized as a legitimate alternative form of programmed cell death that occurs via the defective of autophagy. It is well known that Beclin 1 is a key initiator of autophagic process that mediates the autophagic cell death in cardiomyocytes^19^. In our study, the knockdown of Beclin 1 attenuated H_2_O_2_-induced cell death in cardiomyocytes (Supplementary Fig. 2a). In addition, autophagy inhibitor 3-methyladenine (3-MA) treatment attenuated H_2_O_2_-induced cell death (supplementary Fig. 2b). These results confirm that H_2_O_2_ induces autophagic cell death in cardiomyocytes. The knockdown of myocardin remarkably reduced cell death induced by H_2_O_2_ (Fig. 1f). Taken together, these results suggest that myocardin mediates oxidative stress induced activation of autophagic cell death in cardiomyocytes.

Next, we examined the role of myocardin in the pathogenesis of myocardial infarction using experimental animal model. In mice with myocardial I/R injury, the expression level of myocardin was significantly increased compared to control mice (Supplementary Fig. 2c). The silencing of expression of myocardin by adenoviral dependent delivery of siRNA in vivo (Supplementary Fig. 2d) attenuated autophagy (Supplementary Fig. 3a–c and Fig. 1g). In addition, in vivo administration of myocardin siRNA significantly reduced myocardial infarction size (Fig. 1h) in the heart with I/R injury. To confirm that myocardin mediates autophagic cell death, we administered Beclin 1-siRNA along with adenovirus expressing myocardin and induced myocardial I/R in mice. The overexpression of myocardin augmented I/R-induced myocardial infarction sizes, which was inhibited by knockdown of Beclin 1 (supplementary Fig. 3d), which indicates that myocardin activates autophagy-dependent cell death in cardiomyocytes during I/R injury. The silencing of myocardin significantly protected the myocardial function from I/R injury (Supplementary Fig. 3e, f). Collectively, these data suggest that myocardin contributes to the induction of autophagic process and cardiomyocyte cell death in the heart during I/R injury.

Myocardin acts as a smooth muscle and cardiac muscle-specific transcriptional activator that transactivates the expression of many genes^13^. To understand the mechanisms of autophagy regulation by myocardin in the heart, we examined whether myocardin controls the expression of key molecules (Atg5, Atg7, Atg10, Beclin 1,etc) involved in the execution of autophagic process. The overexpression of myocardin in cultured cardiomyocytes remarkably increased the level of Beclin 1 mRNA, but it did not affect the expression of other autophagy-related genes such as Atg5, Atg7, Atg10 and Atg12 (Supplementary Fig. 4a–e). These results indicate that Beclin 1 might be a potential target of myocardin in the cascade of autophagy.

### p53 activates myocardin transcription and expression

**Fig. 2:**
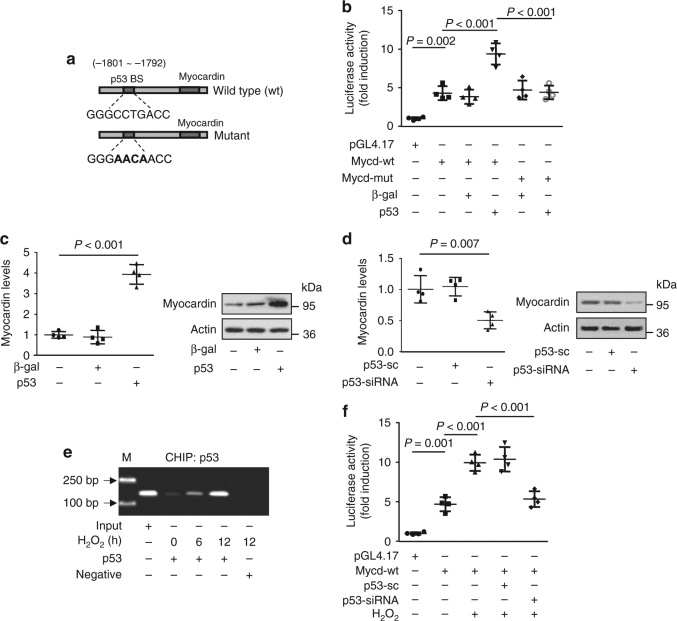
Myocardin is a transcriptional target of p53. **a** Mouse myocardin promoter region contains a potential p53-binding site. **b** p53 promotes myocardin promoter activity. Cardiomyocytes were treated with the adenoviral β-gal or p53, the constructs of the empty vector (pGL-4.17), the wild-type promoter (Mycd-wt) or the promoter with mutations in the binding site (Mycd-mut) respectively. Luciferase activity was assayed. *n* = 4. **c** p53 induces the increase of myocardin expression levels. Cardiomyocytes were infected with adenoviral β-gal or p53. Myocardin expression was analyzed by qRT-PCR (left panel) and immunoblot (right panel). *n* = 4. **d** Knockdown of p53 reduces the myocardin expression. Cardiomyocytes were infected with adenoviral p53-siRNA or p53-sc. Myocardin levels were analyzed by qRT-PCR (left panel) and immunoblot (right panel). *n* = 4. **e** ChIP analysis of p53 binding to the promoter of myocardin. Cardiomyocytes were treated with H_2_O_2_ at indicated time. CHIP was performed with p53 or β-actin (negative) antibody. **f** Knockdown of p53 inhibits the increase of myocardin promoter activity induced by H_2_O_2_. Cardiomyocytes were treated with the adenoviral p53-siRNA or p53-sc, the constructs of the empty vector (pGL-4.17), or the wild-type promoter (wt), and then were treated with H_2_O_2_. Luciferase activity was assayed. *n* = 4

To determine the upstream activator of myocardin expression during autophagy, we analyzed the promoter region of mouse myocardin and found that myocardin possesses potential binding site of p53 (Fig. 2a), which raises the possibility that myocardin could be a direct target of p53. To examine this, we constructed luciferase vectors consisting of wild-type or mutant myocardin and transfected cells. The luciferase assay showed that p53 stimulated the wild-type myocardin promoter activity as indicated by increased luciferase activity, while mutations in the p53-binding site inhibited the interaction of p53 with myocardin promoter as indicated by significantly reduced level of luciferase activity (Fig. 2b). To confirm this result, we enhanced or silenced the expression of p53 using adenoviral vector or p53 specific siRNA respectively and assessed the expression of myocardin in cultured cardiomyocytes. The enforced expression of p53 (Supplementary Fig. 5a) significantly increased the expression of myocardin (Fig. 2c), whereas p53 knockdown (Supplementary Fig. 5b) exhibited a significant decrease in the expression level of myocardin (Fig. 2d). The ChIP assay revealed that p53 is bound to the myocardin promoter under the normal physiological condition in cardiomyocytes, and H_2_O_2_ treatment led to an increase in the association of p53 with myocardin promoter (Fig. 2e). The CHIP assay in the heart with myocardial I/R injury showed that the binding of p53 to the myocardin promoter in the area-at-risk was highly increased compared to that in the remote regions of myocardium with IR injury (Supplementary Fig. 5c, d). These results confirm that myocardin is a potential transcriptional target of p53. By using luciferase reporter system, we observed that H_2_O_2_ induced the elevation of myocardin promoter activity in cardiomyocytes and knockdown of p53 attenuated the increase of myocardin promoter activity (Fig. 2f). These data indicate that p53 can transcriptionally activate myocardin expression.

### Knockdown of p53 inhibits autophagy in vitro and in vivo

**Fig. 3:**
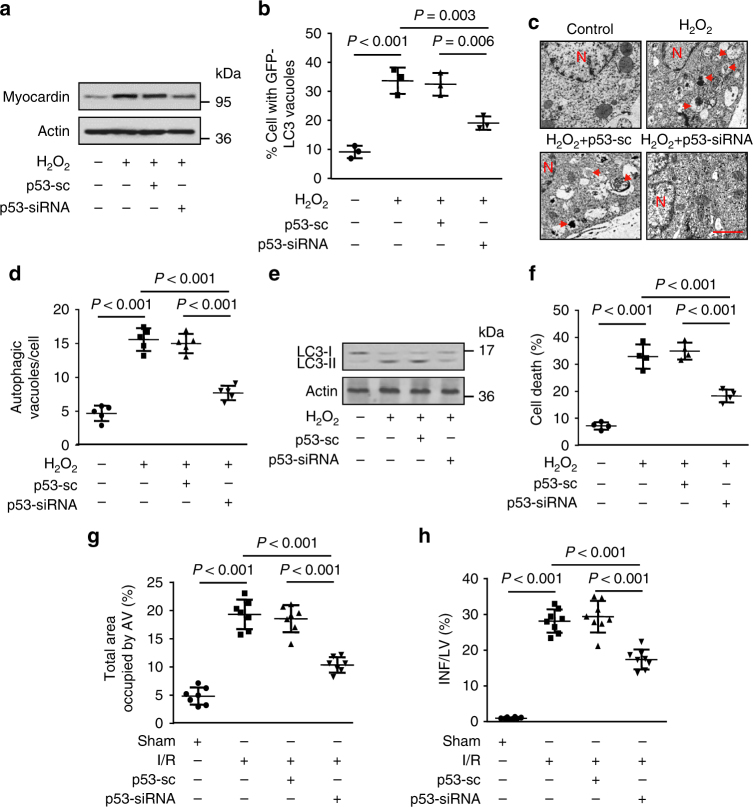
Knockdown of p53 inhibits autophagy in vitro and in vivo. **a** Knockdown of p53 suppresses H_2_O_2_-induced myocardin expression. Cardiomyocytes were infected with adenoviral p53-siRNA or p53-sc. Twenty-four hours after infection, cells were treated with H_2_O_2_. Myocardin levels were analyzed by immunoblot. **b** Knockdown of p53 inhibits punctate accumulations of GFP-LC3. Cardiomyocytes were infected with adenoviral GFP-LC3, p53-siRNA and or p53-sc. Twenty-four hours after infection cells were treated with H_2_O_2_. The percentage of cells with GFP-LC3 puncta was quantified. *n* = 3. **c**, **d** p53 knockdown reduces autophagic vacuoles. Cardiomyocytes were treated as described in (**a**). Representative EM images were shown (**c**). Bar = 2 µM. The arrows depict autophagosomes, and the nucleus is denoted by N. Quantification of autophagic vacuoles was shown in (**d**). *n* = 5. **e** Representative immunoblot for conversion of LC3-I to LC3-II. Cardiomyocytes were treated as described in (**a**). The positions of LC3-I and LC3-II are indicated. **f** p53 knockdown reduces H_2_O_2_-induced cell death. Cardiomyocytes were treated as described in (**a**). Quantitation of cell death is shown. *n* = 4. **g** Knockdown of p53 reduces autophagy in vivo. Mice were injected with adenoviral p53-siRNA or p53-sc as described in methods, and then were subjected to 45 min ischemia and 3 h reperfusion (I/R). Quantification of autophagic vacuoles in the area-at risk was shown. *n* = 7. **h** p53 knockdown reduces I/R-induced myocardial infarction. Mice were treated as described in (**g**). The infarct sizes were quantified. LV left ventricle, INF infarct area. *n* = 8

Next, we tested the functional role of p53 in autophagy. In cultured cardiomyocytes, knockdown of p53 effectively reduced H_2_O_2_ induced increase of myocardin expression (Fig. 3a) and accumulation of GFP-LC3-II punctate (Fig. 3b). The results of electron microscopic analysis showed that p53 knockdown significantly reduced H_2_O_2_-induced accumulation of autophagic vesicles in cultured cardiomyocytes (Fig. 3c, d). In addition, the knockdown of p53 attenuated H_2_O_2_ induced upregulation of LC3-II expression (Fig. 3e) and cell death (Fig. 3f). On the other hand, overexpression of p53 augmented H_2_O_2_-induced cell death in cardiomyocytes, whereas 3-MA treatment remarkably inhibited the effect of p53 on H_2_O_2_-induced cell death (Supplementary Fig. 5e), which indicates that p53 induces autophagic cell death. In animal model of I/R injury, the delivery of p53-siRNA (Supplementary Fig. 5f) attenuated the accumulation of autophagosomes (Fig. 3g) in response to I/R injury. The accumulation of autophagosomes is mainly due to either the overactivation of autophagic flux or the blockage of downstream of autophagic vacuole processing. We thus measured the autophagic flux in the presence of lysosomal inhibitor bafilomycin A1 (BafA1), which inhibits fusion of autophagosome and lysosome. Treatment of I/R- injured mice with BafA1 resulted in a significant increase in LC3-II levels compared to untreated controls, which indicates that I/R enhanced cardiac autophagic flux. The administration of p53 siRNA significantly decreased the conversion of LC3-I to LC3-II in I/R injury even in the presence of BafA1 (Supplementary Fig. 5g). These results demonstrate that p53 is involved in activation of cardiac autophagic flux in the heart during ischemic injury and knockdown of p53 can attenuate this process. In addition, p53-siRNA significant blocked the I/R injury induced increase in the size of myocardial infarction (Fig. 3h). Taken together, these data suggest that p53 contributes to the activation of autophagy signal and cardiomyocyte cell death in the heart.

### CAIF blocks p53-mediated myocardin transcription

**Fig. 4:**
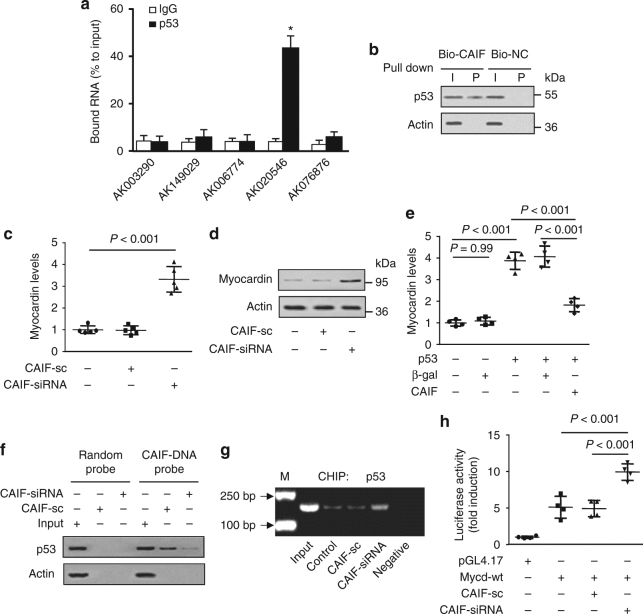
CAIF binds to p53 and blocks p53-mediated myocardin transcription. **a** The qRT-PCR showing lncRNA enrichment by an anti-p53 antibody. Cardiomyocytes were subjected to RIP assay using an anti-p53 antibody or IgG. IP-enriched RNA was then analyzed by qRT-PCR. *n* = 4. **b** Western blot showing p53 protein interaction with CAIF. RNA pull-down assay was performed in cardiomyocytes using biotin-labeled CAIF probe (Bio-CAIF) and negative control probe (Bio-NC). **c**, **d** Knockdown of CAIF increases myocardin expression. Cardiomyocytes were infected with adenovirus CAIF-siRNA or CAIF-sc. Myocardin mRNA levels were analyzed by qRT-PCR (**c**). *n* = 5. Myocardin protein levels were analyzed by western blot (**d**). **e** CAIF inhibits p53-mediated myocardin expression. Cardiomyocytes were infected with adenovirus p53, CAIF or β-gal. Forty-eight hours after infection myocardin levels were analyzed by qRT-PCR. *n* = 4. **f** Knockdown of CAIF decreases its interaction with p53. Cardiomyocytes were infected with adenovirus CAIF-siRNA or CAIF-sc. Forty-eight hours after infection RNA pull-down assay was performed and p53 protein was detected by western blot. **g** CAIF knockdown enhances the binding of p53 to the promoter of myocardin. Cardiomyocytes were infected with adenovirus CAIF-siRNA or CAIF-sc. Forty-eight hours after infection CHIP was performed with p53 antibody. **h** Knockdown of CAIF causes the increase of myocardin transcriptional activity. Cardiomyocytes were treated with the adenoviral CAIF-siRNA or CAIF-sc, the constructs of the empty vector (pGL-4.17) and the wild-type myocardin promoter (Mycd-wt). Luciferase activity was assayed. *n* = 4

To investigate the underlying mechanism of p53-mediated regulation of myocardin and autophagy in cardiomyocytes, we made attempt to identify p53-interacting lncRNA, which has been reported to play important roles in normal physiology as well as in many cardiac diseases. First, we screened the expression of some lncRNAs, which are highly expressed in the heart, using lncRNA array method performed by Fantom project (Supplementary Table 1). We detected the expression levels of lncRNAs in cardiomyocytes using qRT-PCR. Among those lncRNAs, five lncRNAs were substantially decreased upon H_2_O_2_ treatment (Supplementary Table 1). To identify which lncRNA is involved in the regulation of p53, we performed RNA immunoprecipitation (RIP) experiments. The results showed that p53 antibody significantly enriched with AK020546, which we named as CAIF, in cultured primary cardiomyocytes (Fig. 4a) as well as in vivo (Supplementary Fig. 6a). This result indicates that there is a strong interaction between CAIF and the p53 protein. To validate their interaction, we generated a biotinylated probe of CAIF and utilized for RNA pull-down assay. The results showed that CAIF probe strongly bound with p53 in cardiomyocytes (Fig. 4b) and in the mice heart tissue (Supplementary Fig. 6b), which confirms that CAIF is able to directly bind with p53. To examine the p53-binding region of CAIF, we performed deletion-mapping experiments with truncated CAIF. The RNA pull-down experiments with truncated CAIF showed that the 209 nt region at the 3′-end of CAIF is required for the specific interaction with p53 (Supplementary Fig. 6c).

As the CAIF directly interacts with p53, we next examined whether CAIF is involved in the regulation of p53-dependent expression of myocardin. We detected that CAIF is distributed in different cell types including fibroblasts and endothelial cells in the heart, which reveals that the expression of CAIF is not specific to cardiac myocytes (Supplementary Fig. 7a). In cultured primary cardiomyocytes, the knockdown of CAIF (Supplementary Fig. 7b) significantly increased the levels of myocardin mRNA and protein (Fig. 4c, d), while overexpression of CAIF (Supplementary Fig. 7c) significantly decreased the expression of myocardin (Supplementary Fig. 7d, e). Similarly, the silencing of CAIF with siRNA (Supplementary Fig. 7f) remarkably enhanced the myocardin level (Supplementary Fig. 7g). In addition, our data showed that the overexpression of CAIF counteracted the promotive effect of p53 on myocardin expression (Fig. 4e). Next, we sought to explore the mechanism by which CAIF inhibits p53-mediated myocardin expression. For this experiment, we synthesized biotinylated DNA probe complementary to CAIF and RNA pull-down assay was carried out with biotinylated DNA probe. Then, the western blot analysis for p53 was done. Our results showed that CAIF knockdown caused a significant decrease in the binding of p53 (Fig. 4f). The CHIP assay results demonstrate that CAIF knockdown enhanced the binding of p53 to the promoter of myocardin (Fig. 4g) and it significantly increased the myocardin transcriptional activity (Fig. 4h). We also found that the binding of CAIF to p53 is remarkably decreased in the heart with I/R injury (Supplementary Fig. 7h), which might lead to increased association of p53 with myocardin promoter as shown in Supplementary Fig. 5d. Taken together, our data suggest that CAIF binds to p53 in vivo, and this interaction blocks p53-mediated myocardin transcription.

### CAIF blocks autophagic signal in cardiomyocytes and in vivo

**Fig. 5:**
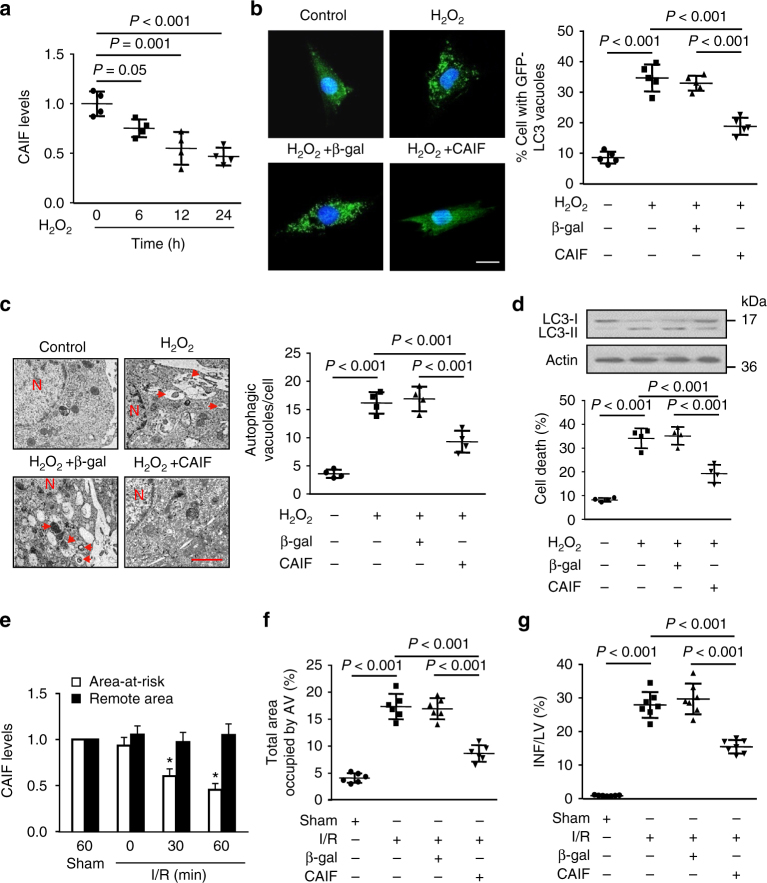
CAIF inhibits autophagy in cardiomyocytes and in vivo. **a** H_2_O_2_ induces a decrease in CAIF levels. Cardiomyocytes were treated with H_2_O_2_ at indicated time. CAIF were analyzed by qRT-PCR. *n* = 4. **b** CAIF inhibits punctate accumulations of GFP-LC3. Cardiomyocytes were infected with adenoviral GFP-LC3, CAIF or β-gal. Twenty-four hours after infection cells were treated with H_2_O_2_. Representative images show GFP-LC3 staining (left panel), Bar = 20 μm. The percentage of cells with GFP-LC3 puncta was quantified in (right panel). *n* = 5. **c** CAIF reduces autophagic vacuoles in cardiomyocytes. Cells were infected with adenoviral CAIF or β-gal. Twenty-four hours after infection cells were treated with H_2_O_2_. Representative EM images were shown (left panel). Bar = 2 µM. The arrows depict autophagosomes, and the nucleus is denoted by N. Quantification of autophagic vacuoles was shown in right panel. *n* = 4. **d** CAIF inhibits the conversion of LC3-I to LC3-II and reduces cell death in response to H_2_O_2._ Cardiomyocytes were treated as described in (**c**). Representative immunoblot for conversion of LC3-I to LC3-II was shown (upper panel). Quantitation of cell death was shown (low panel). *n* = 4. **e** Mice were subjected to ischemia at indicated time and 3 h reperfusion (I/R). Area-at-risk and the remote zone were prepared for qRT-PCR analysis of CAIF levels. *n* = 6. *p < 0.05 vs. sham. **f** CAIF reduced autophagy upon I/R injury. Mice were injected with CAIF or β-gal as described in methods, then were subjected to 45 min ischemia and 3 h reperfusion (I/R). Quantification of autophagic vacuoles in the area-at risk was shown. *n* = 6. **g** CAIF reduces myocardial infarction upon I/R in vivo. Mice were treated as described in (**f**). The infarct sizes were quantified. LV left ventricle, INF infarct area. *n* = 7

We next investigated the function of CAIF during autophagic cell death in cardiomyocytes. In cardiomyocytes exposed to H_2_O_2_, the level of CAIF was significantly decreased along with increased level of autophagy (Fig. 5a). The overexpression of CAIF significantly inhibited H_2_O_2_-induced autophagy (Fig. 5b–d and Supplementary Fig. 8a). To determine the functional relevance of the interaction between CAIF and p53 and the induction of autophagy, we observed the ability of different truncated versions of CAIF to regulate H_2_O_2_-induced autophagy. Our results found that the deletion of 209 nt region at the 3’ end of CAIF failed to inhibit autophagy induced by H_2_O_2_ (Supplementary Fig. 8b). This result indicates that the 209 nt fragment at the 3′-end of CAIF alone is sufficient to inhibit autophagy. In addition, the overexpression of CAIF remarkably inhibited H_2_O_2_-induced cell death in cardiomyocytes (Fig. 5d, lower panel). In contrast, the knockdown of CAIF significantly increased H_2_O_2_-induced cell death, which was inhibited by treatment with 3-MA (Supplementary Fig. 8c). This data suggest that the expression of CAIF is required to block autophagic cell death in cardiomyocytes.

In mice model of I/R injury, the expression of CAIF was significantly decreased (Fig. 5e). The administration of CAIF (Supplementary Fig. 9a) attenuated autophagy (Fig. 5f and Supplementary Fig. 9b) and infarction size induced by I/R injury in mice (Fig. 5g). The echocardiography results indicate that CAIF administration improved I/R injury induced dysregulation in the heart function (Supplementary Fig. 9c, d). Together, these data demonstrate that the expression of CAIF is essential to inhibit autophagy induced cardiomyocyte cell death and cardiac dysfunction caused by ischemic injury.

### CAIF regulates autophagy through controlling p53 and myocardin

**Fig. 6:**
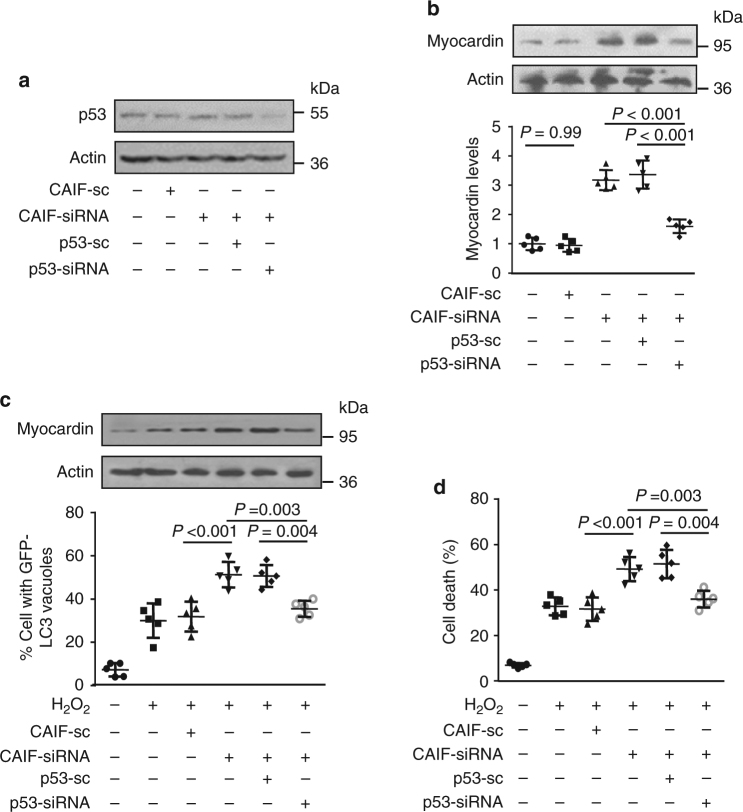
CAIF exerts its autophagic effect through p53 and myocardin. **a**, **b** Knockdown of p53 reduces the promoting effect of CAIF knockdown on myocardin expression. Cardiomyocytes were infected with adenoviral CAIF-siRNA or CAIF-sc, p53-siRNA or p53-sc. p53 expression levels were analyzed by immunoblot (**a**). Myocardin expression levels were analyzed by immunoblot (**b**, upper panel) and by qRT-PCR (**b**, low panel). *n* = 5. **c**, **d** Knockdown of p53 attenuates the promoting effect of CAIF knockdown on autophagy and cell death induced by H_2_O_2_. Cardiomyocytes were infected with adenoviral CAIF-siRNA or CAIF-sc, p53-siRNA or p53-sc, and then exposed to H_2_O_2_. Myocardin levels were analyzed by immunoblot (**c**, upper panel). Autophagy was assessed by GFP-LC3 staining. The percentage of cells with GFP-LC3 puncta was quantified in (**c**, low panel). *n* = 5. Quantitation of cell death is shown in (**d**). *n* = 5

Next, we performed the experiments to confirm whether myocardin is a downstream target of CAIF during autophagy. We detected myocardin expression and the results showed that knockdown of p53 (Fig. 6a) reduces the promoting effect of CAIF knockdown on expression of myocardin (Fig. 6b). The knockdown of CAIF significantly enhanced H_2_O_2_-induced myocardin expression, but co-transfection of p53 siRNA significantly attenuated the effect of CAIF knockdown on myocardin expression (Fig. 6c, upper panel). We also found that knockdown of CAIF aggravated H_2_O_2_-induced autophagy and cell death in cardiomyocytes. However, the silencing of p53 expression attenuated the effects of CAIF knockdown on autophagy (Fig. 6c, low panel) and cell death (Fig. 6d). Together, these data suggest that CAIF exerts its inhibitory effect on autophagy and cell death through regulating p53 activity and myocardin expression.

## Discussion

Autophagy is generally regarded as an adaptive response to stress that helps to maintain the intracellular homeostasis by removing damaged proteins and intracellular organelles. The autophagy can protect cells from different kinds of injuries^20,21,22^. However, excessive or defective autophagy is detrimental to cells by causing large scale accumulation of autophagic products such as autophagosomes as well as degradation of vital proteins and organelles involved in survival. Current studies suggest that autophagy is either protective or detrimental to the cardiac tissue depends upon the degree of induction and duration of the injury. Under ischemic condition, the acceleration of autophagy during reperfusion can cause damage to cardiomyocytes and it enhances cardiac cell death^23^. It is well known that oxidative stress and activation of autophagy are major players in the cardiomyocyte cell death and cardiac injury associated heart dysfunction^24^. In the present study, we demonstrate that oxidative stress induced increase of p53 promotes the expression of myocardin, which is involved in the activation of autophagic process. The overactivation of autophagy by p53-myocardin signaling leads to cardiomyocyte cell death and acceleration of ischemic injury. Most interestingly, we found that a cardiac specific expression of an lncRNA, named as CAIF, can inhibit p53-dependent expression of myocardin and autophagy induction in cardiomyocytes. However, the depletion of CAIF expression in cardiomyocytes during I/R injury leads to autophagic cell death and cardiac dysfunction. This is the first study to illustrate that p53-myocardin dependent activation of autophagy is involved in cardiomyocyte cell death and CAIF play indispensible role in governing this molecular axis to control autophagic process.

LncRNAs are critical regulator of various cellular processes. They play an important role in biological functions and the alterations in expression is well connected to development and progression of various diseases^25–28^. Several lncRNAs have been reported to participate in the regulation of autophagy^29,30^. However, very few studies demonstrate the regulatory functions of lncRNAs in autophagy in the heart. In the present study, we have identified a new lncRNA (CAIF), which has the capability to regulate autophagy in cardiomyocytes. Our results found that enforced expression of CAIF inhibits autophagic cell death and attenuates myocardial infarction sizes in the I/R heart. Our results reveal that CAIF could be a potential therapeutic tool to prevent the defective autophagy-mediated loss of cardiac myocytes as well as for the treatment of myocardial infarction and heart failure.

LncRNAs regulates the expression and activity of target molecules through different mechanisms including epigenetic modification^31–33^, protein degradation^34^, RNA stability, translation and splicing and trafficking^8^. LncRNAs also participate in the regulation of RNA transcription. For example, the lncRNA Xist mediated transcriptional silencing by recruiting polycomb repressive complex 2 (PRC2) across the X chromosome^35^. The H19 RNA recruits MBD1, interacts with methyltransferases, induces repressive histone modification in the target genes and finally controls target genes expression^36^. The lncRNA HOTAIR mediates the interaction between Snail and enhancer of zeste homolog 2 (EZH2), and represses downstream targets^37^. Likewise, the newly identified lncRNA, CAIF, regulates the expression of myocardin. However, the regulatory mechanism of gene expression is differing from other lncRNAs. Surprisingly, lncRNA CAIF directly interacts with p53, a transcription factor and blocks its binding to promoter region of myocardin. Our finding indicates that lncRNA can interfere gene expression by forming complex with transcription factors and inhibiting their activity. In addition, our study suggests that lncRNAs could have diverse mechanism for regulating gene expression beyond its well defined epigenetic regulatory functions. Thus, further characterization of lncRNA signaling pathways and regulatory mechanisms of lncRNA will provide a better understanding about lncRNA-mediated gene regulation. Currently, it is unknown about the factors associated with the suppression of level of CAIF under oxidative stress condition as well as in I/R injury. The depletion of CAIF under I/R condition could be either due to inhibition of its expression or increased degradation. Further detailed studies are warranted to address this phenomenon.

Some previous studies show that p53 suppresses autophagy and inhibition of p53 degradation prevents the activation of autophagy in HCT116 colon cancer cells and HeLa cell^12^. Other studies suggest that p53 is able to induce autophagy^38–40^. In consistent with these reports, our data show that p53 promotes autophagy. Our results demonstrate that the inhibition of p53 with siRNA attenuates autophagic cell death and myocardial infarction sizes in the heart with I/R injury, while overexpression of p53 enhanced H_2_O_2_-induced autophagic cell death and inhibition of autophagy with 3-MA blocked this response in cardiomyocytes. These findings confirm the relationship between p53 and autophagy in induction of cell death and it is of great importance to understand many physiological and pathological processes. As the dysregulation of p53 activity and autophagy play crucial role in the progression of cardiac injury, our study strengthen the notion that p53 mediated sustained activation of autophagy is detrimental to cells and tissue function.

p53 is a sequence-specific DNA-binding transcription factor and many genes can be regulated by p53 at transcriptional level. Our current results demonstrate that myocardin functions as a downstream target of p53 in regulating autophagy. p53 promotes myocardin expression and regulates cardiomyocyte autophagy. Several previous studies suggest that p53 can regulate myocardin. p53 regulates smooth muscle cells differentiation through targeting myocardin^41^. In VSMC, the treatment of antioxidant resveratrol activates p53 signaling and downregulated the transcription of myocardin^42^. Given the fact that the activity and response of transcription factors vary depends on their upstream activators and it also differs in various cell types. In this context, it is possible that p53 activates myocardin expression in cardiomyocytes but it has inhibitory function in differentiating smooth muscle cells. A further detailed study will be required to delineate this variation of p53-dependent expression of myocardin in different cell types.

It is well known that myocardin has important functions in the heart and contributes to the cardiac growth, chamber maturation, and embryonic survival. Myocardin is involved in cell proliferation, migration, and myogenesis by interacting with serum response factor (SRF)^13,43^. It acts as a smooth muscle and cardiac muscle-specific transcriptional activator and transactivates the expression of atrial natriuretic factor (ANF), myosin light chain (MLC)-2V, and α-MHC genes, as well as the *Nkx*2.5 gene^13^. Our study for the first time demonstrates the function of myocardin in autophagy induction. Our data show that myocardin participates in the regulation of cardiomyocytes autophagy upon H_2_O_2_ or I/R treatment. In addition, we also demonstrate that myocardin induces an increased expression of Beclin 1, which is required for the execution of autophagy, indicating that there is a cross-talk between myocardin and Beclin 1. We will attempt to further clarify the relationship between myocardin and Beclin 1 in the autophagic machinery in future studies.

## Methods

### Treatment and culture of cardiomyocytes

We isolated cardiomyocytes from 1- to 2-days-old mice. First, hearts were washed and minced in HEPES-buffered saline solution after dissected from the mice. Tissues were then dispersed in a series of incubations at 37 °C in HEPES-buffered saline solution containing 1.2 mg/ml pancreatin and 0.14 mg/ml collagenase. Lysates were collected and centrifuged at 200 × *g* for 5 min. After centrifugation, precipitated cells were re-suspended in Dulbecco’s modified Eagle medium/F-12 (GIBCO) supplemented with 5% heat-inactivated horse serum, ascorbate (0.1 mM), insulin-transferring-sodium selenite media supplement (Sigma, St. Louis, MO), penicillin (100 U/ml), streptomycin (100 μg/ml), and bromodeoxyuridine (0.1 mM). The cells were pre-plated at 37 °C for 1 h. Dissociated cells were then diluted to 1 × 10^6^ cells/ml and plated in 10 μg/ml laminin-coated different culture dishes. In cell death assay, we used 200 µM H_2_O_2_ to treat isolated cardiomyocytes in vitro. Cells were then stained by Trypan Blue. And we counted the numbers of Trypan Blue-positive and Trypan Blue-negative cells using hemocytometer and determined the rate of cell death.

Autophagic flux was analyzed in cardiomyocytes infected with adenovirus expressing tandem RFP-GFP-LC3 and then treated as indicated. The cells were washed with PBS, fixed with 4% paraformaldehyde and visualized using a Zeiss LSM-510 confocal laser-scanning microscope. LC3 green and red dots were quantified using the ImageJ software. A minimum of 50 cells were scored for each condition. An observer blinded to sample identity assessed the autophagic flux. The raw data for measurement of autophagic flux are shown in Supplementary Data set 1.

### Adenoviral constructions and infection

We use mouse cDNA as the template to obtain the full length of CAIF by PCR. The forward primer was 5′-TCGTGAATTTTGTCAGTTTTGTGATATCC-3′; the reverse primer was 5′-AGGTAAGTTTAACTGGTCAGGAAATAAAC-3′. Mouse myocardin cDNA was purchased from Origene. We use the Adeno-X™ expression system (Clontech) to construct adenoviruses harboring the CAIF, myocardin, and β-galactosidase (β-gal), respectively. The mouse CAIF-siRNA target sequence is 5′-GGGACGCTTGTGCAAACTT-3′. A scramble form was used as a control, 5′-GTATCGCGAGTAGTCAGTC-3′. The mouse myocardin siRNA target sequence is 5′-GCACACGAAGGCCATTCTT-3′. A scramble form was used as a control, 5′-CTCAGACTGCAGTACGACT-3′. Beclin 1 siRNA target sequence is 5′-GATCCTGGACCGGGTCACC-3′; the scramble sequence is 5′-CCTAGGCTCACGTGACGCG-3′. We use the pSilencer™ adeno 1.0-CMV System (Ambion) to generate adenoviruses carrying CAIF-siRNA, myocardin siRNA, Beclin 1 siRNA and their scramble forms according to the Kit’s instructions. And HEK293 cells were used to amplify all constructs.

### Immunoblot

Immunoblot was performed according to the following procedure. Cells were lysed in the lysis buffer for 1 h at 4 °C. Components of the lysis buffer are 20 mmol/L Tris pH 7.5, 2 mmol/L EDTA, 3 mmol/L EGTA, 2 mmol/L dithiothreitol (DTT), 250 mmol/L sucrose, 0.1 mmol/L phenylmethylsulfonyl fluoride, 1% Triton X-100, and a protease inhibitor cocktail is also included. Samples were loaded into the wells of 10 or 12% SDS-PAGE, and transferred to nitrocellulose membranes after electrophoresis. Blots were probed using the primary antibodies. The anti-Myocardin antibody (1:500, Abcam, ab203614), anti-p53 antibody (1:500, Abcam, ab131442), anti-Actin antibody (1:2000, Abcam, ab6276), and anti-LC3 antibody (1:500, Abcam, ab192890) were used in this study. After incubated with the primary antibody, we wash the membrane using PBS for four times and added the horseradish peroxidase-conjugated secondary antibodies. Antigen–antibody complexes were visualized by enhanced chemiluminescence. Uncropped blots are shown in Supplementary Figs. 10 and 11.

### Quantitative real-time PCR (qRT-PCR)

CAIF was quantified using SYBR® Green real-time PCR. The sequences of CAIF primers were forward: 5′-CTTCACTCCTGCAAATGTGTT-3′; reverse: 5′-TTATAGTGGGATGGGCAGTTT-3′. qRT-PCR for myocardin was performed as we described^44^. The sequences of myocardin primers were forward: 5′-TCAGTCTTACAGTTACGGCTT-3′; reverse: 5′-GCACTATCCAATTGTTTTCTCG-3′. The sequences of ATG5 primers were forward: 5′-GCGGCCTTTCATCCAGAAGC-3′; reverse: 5′-CGACTGCGGAAGGACAGACT-3′. The sequences of ATG7 primers were forward: 5′-GCCAGGACACCCTGTGAACT-3′; reverse: 5′-CCAAGGCAGCGTTGATGACC-3′. The sequences of ATG10 primers were forward: 5′-CAGCGGTGGCAGAAGTGATT-3′; reverse: 5′-TGTTCCTGCTGGGTGATGGT-3′. The sequences of ATG12 primers were forward: 5′-GACGTTAGAGTGTGCCATTA-3′; reverse: 5′-GTAAATGCCTTCAGTCATCC-3′. The sequences of Beclin 1 primers were forward: 5′-GCACCATGCAGGTGAGCTTC-3′; reverse: 5′-TTTCGCCTGGGCTGTGGTAA-3′. The results were standardized to control values of glyceraldehyde-3-phosphate dehydrogenase (GAPDH). GAPDH forward primer: 5′-TGTGTCCGTCGTGGATCTGA-3′; reverse: 5′-CCTGCTTCACCACCTTCTTGA-3′. We used agarose gel electrophoresis to confirm the specificity of the PCR amplification. The relative expression of different sets of genes was quantified to GAPDH mRNA.

### RNA-binding protein immunoprecipitation (RIP) assay

We performed RIP using a Magna RIP RNA-Binding Protein Immunoprecipitation Kit (Millipore) according to the manufactuer’s protocal. Briefly, cardiomyocytes were harvested by adding RIP lysis buffer and incubated with protein beads and p53 antibody complex overnight at 4 °C. After washing off unbound materials, RNAs binding to p53 were eluted and quantified. We used qRT-PCR to examine certain RNAs co-immunoprecipitated with the p53 antibody.

### RNA pull-down assay

We used mpliScribe™ T7-Flash™ Biotin-RNA Transcription Kit (Epicentre, Madison, WI, USA) to transcribe and purify Biotin-labeled CAIF probe in vitro. The biotin-labeled probe was incubated with cells protein extract for 2 h. Incubating the mixture with streptavidin agarose beads (Invitrogen) at room temperature (RT) for 1 h. After stringent washing with the wash/binding buffer, the retrieved protein was analyzed by western blot.

### Pull-down assay with biotinylated DNA probe

We dissolved the biotinylated DNA probe complementary to CAIF in 500 µl of wash/binding buffer (0.5 M NaCl, 20 mM Tris-HCl, pH 7.5, and 1 mM EDTA). Streptavidin-coated magnetic beads (Sigma) were incubated with the probes at 25 °C for 2 h to generate probe-coated magnetic beads. Cardiomyocyte lysates were incubated with probe-coated beads. After washing with the wash/binding buffer, the protein complexes bound to the beads were eluted, extracted and loaded onto SDS-PAGE for western blot analysis. CAIF pull-down probe, 5′-GCTACTGCACACTCAATTCTGGGAGACC-3′; and random pull-down probe, 5′-TGATGTCTAGCGCTTGGGCTTTG-3′.

### Chromatin immunoprecipitation (ChIP) assay

ChIP assay was performed as follows. In brief, cells were washed with PBS and fixed for 10 min with 1% formaldehyde at room temperature. The cross-linking reaction was quenched using 0.1 M glycine and treated for 5 min. Cells were washed twice with PBS and lysed for 1 h at 4 °C in a lysis buffer. The cell lysates were sonicated into chromatin fragments with an average length of 500 to 800 bp. For ChIPs, the samples were precleared with Protein-A agarose (Roche) for 1 h at 4 °C on a rocking platform. After that, 5 μg specific antibodies were added and rocked for overnight at 4 °C. We used salmon sperm DNA blocked Protein-A agarose to capture immunoprecipitates. The QIAquick Spin Kit (Qiagen) was used to purify DNA fragments. The purified was used as a template and amplified with the following primer sets. For the analysis of p53 binding to the promoter region of myocardin, the oligonucleotides were forward: 5′-TTTCACAGAGTTTCCTCCATG-3′; reverse: 5′-TCCCAGCTCATCAAAGAAGA-3′.The heart samples from mice were subjected to CHIP assay using EpiQuik Tissue Chromatin Immunoprecipitation (ChIP) Kit according to manufacturer’s instructions.

### Construction of mouse myocardin promoter

The myocardin promoter was amplified from mouse genomic DNA by PCR. The forward primer was 5′-TTTCACAGAGTTTCCTCCATG-3′. The reverse primer was 5′-ACCTTCCTTTTTCCCATTCTC-3′. The myocardin promoter luciferase reporter plasmid pGL4.17-Mycd was constructed by ligating the myocardin promoter region into the pGL4.17 vector (Promega). Site-directed mutagenesis in the putative p53-binding site was performed using the QuikChange II XL Site-Directed Mutagenesis Kit (Stratagene). We sequenced the construct to make sure that only the desired mutations had been introduced.

### Luciferase activity assay

Dual-Luciferase Reporter Assay System (Promega) was used to perform luciferase activity assay according to the manufacturer’s instructions. The constructed pGL4.17-Mycd or pGL4.17-Mycd–mut were transfected into cells (150 ng/well) using Lipofectamine 2000 (Invitrogen). Then, those cells were infected with indicated adenovirus. At 48 h after infection, luciferase activity was measured.

### Transmission electron microscopy

Conventional electron microscopy was performed as follows. Cells were fixed with 2.5% glutaraldehyde and then postfixed with 1% osmium tetraoxide. Samples were dehydrated in an ethanol series and embedded in Embed812 resin. The ultrathin sections were cut using a diamond knife. Then, the sections were mounted on copper grids and double-stained with uranyl acetate and lead citrate. The number of autophagic vacuoles was determined for a minimum of 100 cells. Heart ultrastructural analysis was also performed. The observation and microphotographs of the samples were carried out using a FEI Tecnai spirit transmission electron microscope. The investigator who assessed the autophagic vesicle was blinded to the EM sample identity.

### Echocardiographic assessment

After 1 week of the sham or I/R surgery, transthoracic echocardiography was used on mice to assess cardiac structure and function. Echocardiographic parameters were measured. We use M-mode echocardiography in measuring Left ventricular ejection fraction (LVEF) and Left ventricular end-systolic volume (LVESV). Measurements were taken from more than three beats and averaged. Raw data for measurement of cardiac function are shown in Supplementary Data set 1. After in vivo echocardiography study of cardiac function, the mice hearts were collected, weighted and fixed for histological examination.

### Animal experiments

Experiments were performed in male adult C57BL/6 mice (10 weeks old), which were obtained from Institute of Laboratory Animal Science of Chinese Academy of Medical Sciences (Beijing, China). All experiments complied with the guiding principles for the care and use of laboratory animals in Qingdao University and were approved by the Committee for Animal Experimentation. Animals used in experiments had similar body weight. Animals used for statistical analysis had normal physiological index during surgical experiments. Computer-generated random numbers were assigned to each mouse. We allocated mice to each group by chance.

We performed intracoronary delivery of adenoviruses. Mice were anesthetized and intubated, ventilated with a HX-300S animal ventilator. The heart was exposed through a small left anterior thoracotomy. After removal of the pericardial sac, 2 × 10^11^ moi adenoviruses of CAIF, 2 × 10^10^ moi adenoviruses of myocardin-siRNA, 2 × 10^10^ moi adenoviruses of p53-siRNA or 2 × 10^11^ moi adenoviruses of Beclin 1-siRNA were injected, respectively, through a catheter from the apex of the heart left ventricle into the aortic root while the aorta and pulmonary arteries were cross-clamped for 20 s. The chest was then closed after removal of air and blood. Then, mice were allowed to recover. Five days after the injection of adenoviruses, the mice were subjected to I/R surgery. To examine the effects of 3-MA, the mice were injected with 3-MA 1 h before I/R surgery.

Experimental strategies for I/R injury model are as follows. Cardiac I/R in mice was induced by 45 min ischemia, followed by 3 h or 1 week reperfusion. After 3 h of reperfusion, we injected evans blue dye (1 ml of a 2.0% solution; Sigma-Aldrich) into jugular vein into the heart. The ischemic zone and the nonischemic zone was delineated by the dye. The heart was rapidly excised and sectioned serially into 1 mm-thick sections. Then, slices were incubated in 1.0% 2,3,5-triphenyltetrazolium chloride (Sigma-Aldrich) for 15 min at 37 °C to determine the infarct area. The staining was stopped by ice-cold sterile saline and slices were fixed in 10% neutral buffered formaldehyde for another 15 min. The slices were weighed and both sides of each slice were digitally photographed. The areas of infarction (INF) and nonischemic left ventricle (LV) were measured and determined using computerized planimetry (NIH Image 1.57) by an observer blinded to the sample identity. Raw data for measurement of infarction size are shown in Supplementary Data set 1.

To assess induction of autophagy in vivo, adenovirus RFP-GFP-LC3 was administered into the infarct border zone of mice after I/R. Mice were killed 7 days later and GFP and RFP signals were analyzed on frozen sections by confocal microscopy. LC3 green and red dots were quantified using the ImageJ software. Raw data for measurement of autophagy are shown in Supplementary Data set 1. Autophagic flux was assessed by western blot. Bafilomycin A1 (BafA1) was administered to mice by intraperitoneal injection (1.5 mg/kg) 2 h before they were euthanized. To limit circadian variability of autophagy, mice were euthanized at the same time of day.

### Statistical analysis

Each cellular experimental group was repeated for at least three times. And each animal group was repeated for at least five times. The data are expressed as the mean ± SD calculated by GraphPad Prism or SPSS. For multiple comparisons, one-way analysis of variance (ANOVA) followed by Tukey post hoc test was performed. The results were considered statistically significant when *P* < 0.05. All statistical analyses were performed with GraphPad Prism Version 6 (GraphPad Software Inc., SanDiego,CA,USA) and SPSS package (SPSS Inc., Chicago, IL, USA).

### Data availability

All relevant data are provided in this article and its supplementary information files, or are available from the corresponding authors upon reasonable request.

## Electronic supplementary material

Supplementary Information

Description of Additional Supplementary Files

Supplementary Data 1
