# Supplementary material for: LncRNA CAIF inhibits autophagy and attenuates myocardial infarction by blocking p53-mediated myocardin transcription
Source: Nat Commun. 2018 Jan 2;9:29. doi: 10.1038/s41467-017-02280-y (PMC5750208; doi:10.1038/s41467-017-02280-y)
Supplement: Supplementary file 1 — Supplementary Information [file 41467_2017_2280_MOESM1_ESM.pdf]

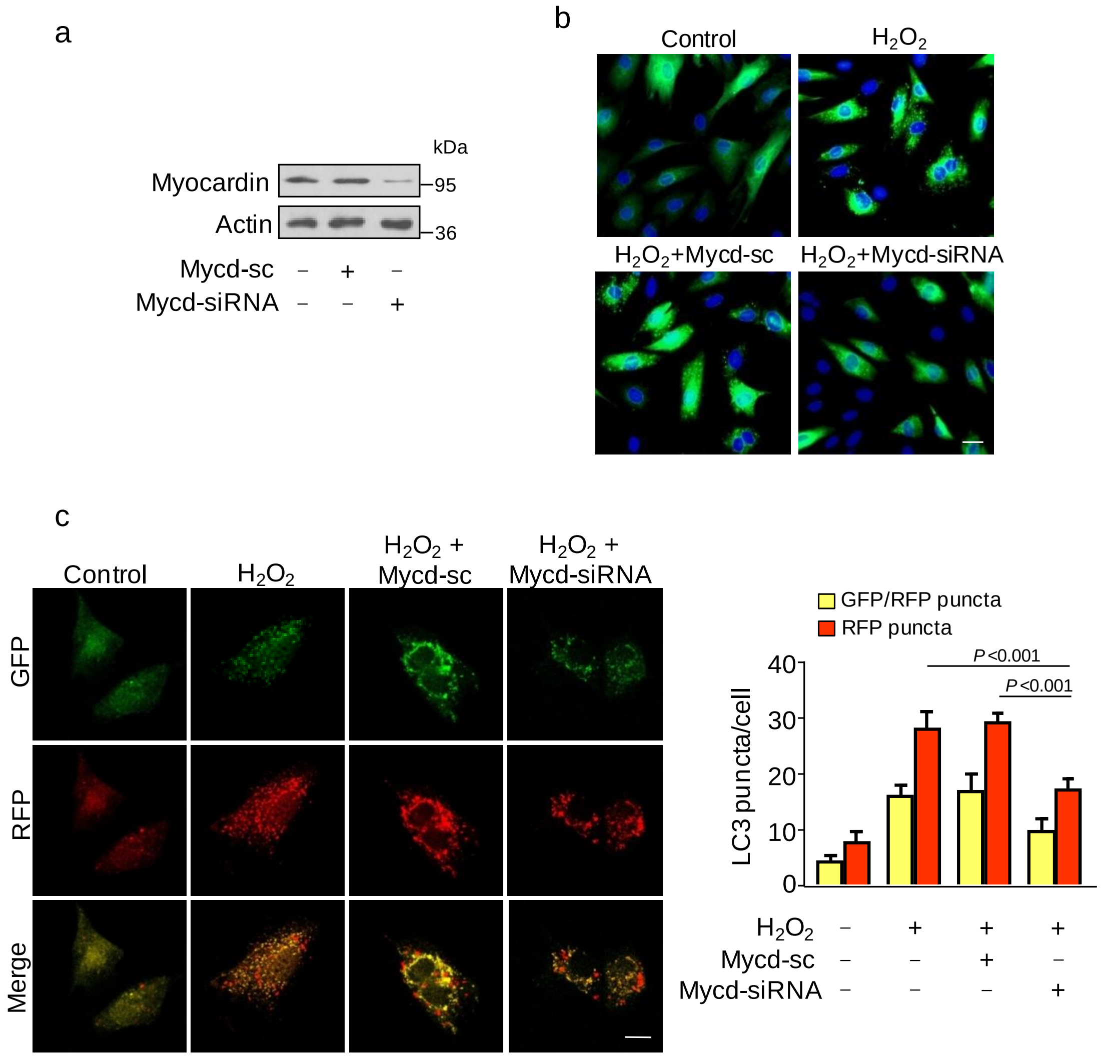

Supplementary Figure 1. Myocardin regulates H_2O_2-induced autophagy. (**a**) Knockdown of myocardin reduces the expression levels of myocardin. Cardiomyocytes were infected with adenoviral Mycd-siRNA or Mycd-sc. 24h after infection myocardin levels were analyzed by immunoblot. (**b**) Knockdown of myocardin inhibits punctate accumulations of GFP-LC3 induced by H_2O_2. Cardiomyocytes cells were infected with adenoviral myocardin siRNA (Mycd-siRNA) or its scramble form (Mycd-sc), then infected with GFP-LC3. 24 h after infection cells were treated with H_2O_2. Representative photos of GFP-LC3 cells were shown. Bar=20μm. (**c**) Myocardin knockdown inhibits autophagy flux. Cardiomyocytes were infected with the RFP-GFP tandem fluorescent-tagged LC3 adenovirus and Mycd-siRNA or Mycd-sc, 24 h after infection cells were treated with H_2O_2. Bar=20μm. Numbers of autophagosomes (yellow puncta) and autolysosomes (red puncta) in each cell were quantified (n=50 cells per group). n=5.

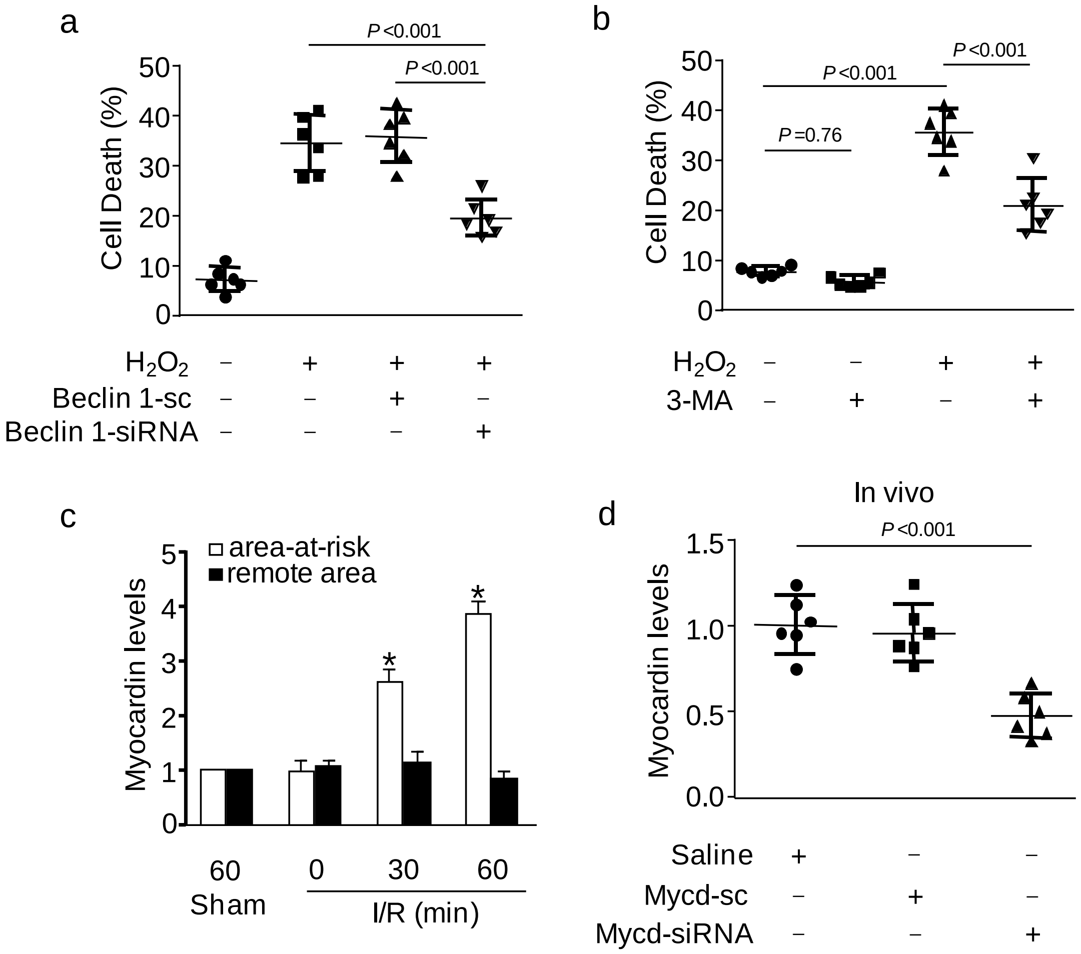

Supplementary Figure 2. The efficacy of myocardin knockdown in vivo. (**a**) Beclin 1 knockdown attenuates H_2O_2-induced cell death. Cardiomyocytes were infected with adenoviral Beclin-siRNA or Beclin-sc. 24 h after infection, cells were treated with H_2O_2. Quantitation of cell death is shown. n=6. (**b**) Cardiomyocytes were treated with H_2O_2 in the presence or absence of 3-MA. 3-MA was preincubated with cells for 1h. Cell death was determined. n=6. (**c**) Mice were subjected to ischemia at indicated time and 3h reperfusion (I/R). Area-at-risk and the remote zone were prepared for qRT-PCR analysis of myocardin levels. n=6. *p<0.05 vs sham. (**d**) Mice were injected with adenovirus Mycd-siRNA or Mycd-sc as described in methods, myocardin levels were analyzed by qRT-PCR. n=6.

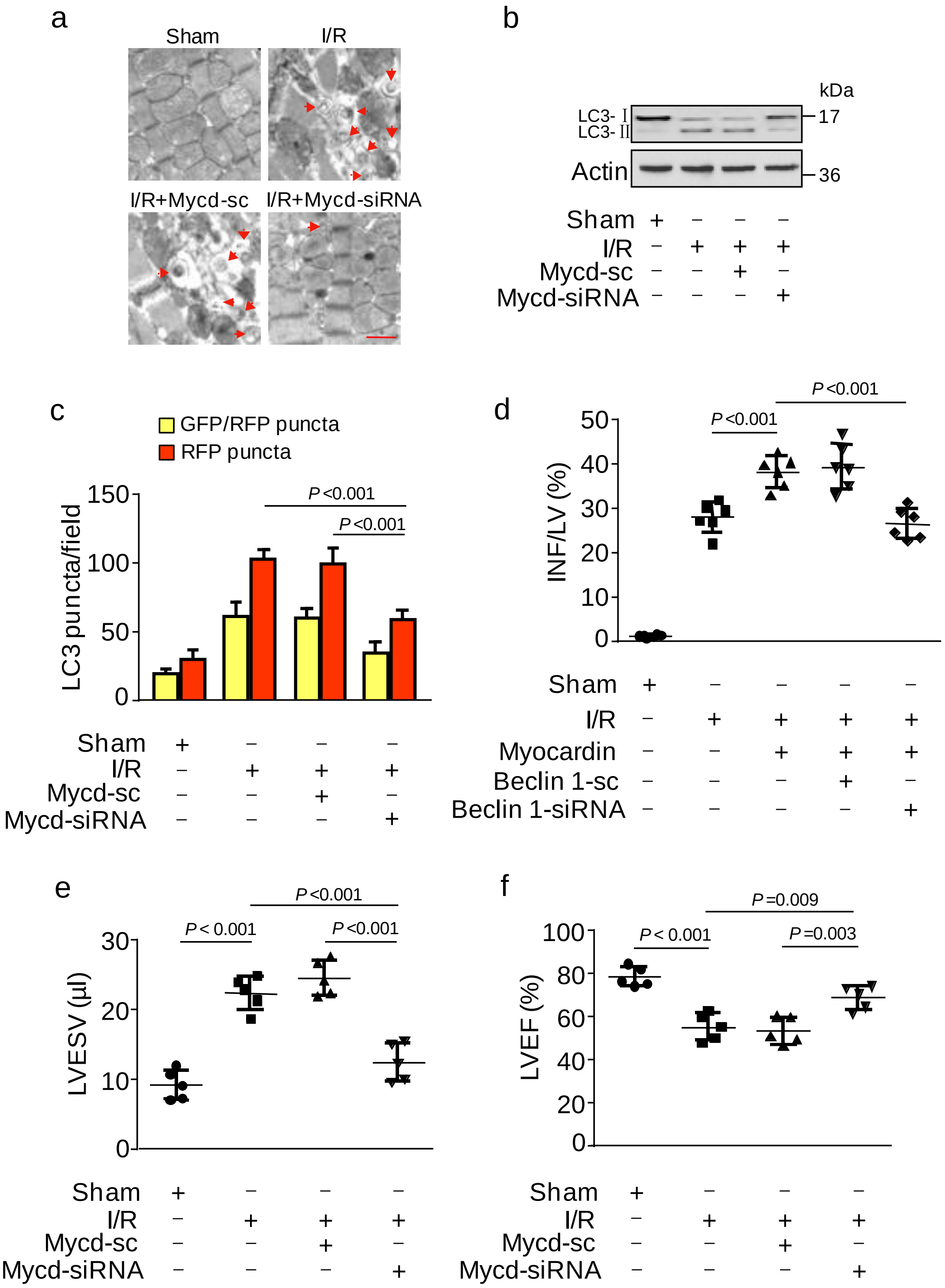

Supplementary Figure 3. Myocardin mediates autophagy in vivo. (**a** and **b**) Mice were injected with adenovirus Mycd-siRNA or Mycd-sc as described in methods, and then were subjected to 45 min ischemia and 3h reperfusion (I/R). (a) Representative electron micrographs (EM) of the area at risk show autophagosomes (arrow). Bar=500nm. (b) LC3 levels were analyzed by immunoblot and the positions of LC3-I and LC3-II are indicated. (**c**) Heart sections of mice injected with adenovirus RFP-GFP-LC3 and Mycd-siRNA or Mycd-sc and subjected to 45 min ischemia and

3h reperfusion. Quantification of GFP and RFP LC3-positive dots per field in the area at risk was shown. n=6 mice per group. (**d**) Myocardin augments myocardial infarction size through promoting autophagy. Mice were injected with adenovirus myocardin, Beclin 1-siRNA or Beclin 1-sc as described in methods, and then were subjected to 45 min ischemia and 3h reperfusion (I/R). Infarct sizes were analyzed. Left ventricle (LV), infarct area (INF). n=6. (**e** and **f**) Mice were injected with adenovirus Mycd-siRNA or Mycd-sc as described in methods, then were subjected to 45 min ischemia and 1 week reperfusion (I/R). Echocardiography was employed to test cardiac function. (e) LVESV, left ventricular end-systolic volume; (f) LVEF, left ventricular ejection fraction. n=5.

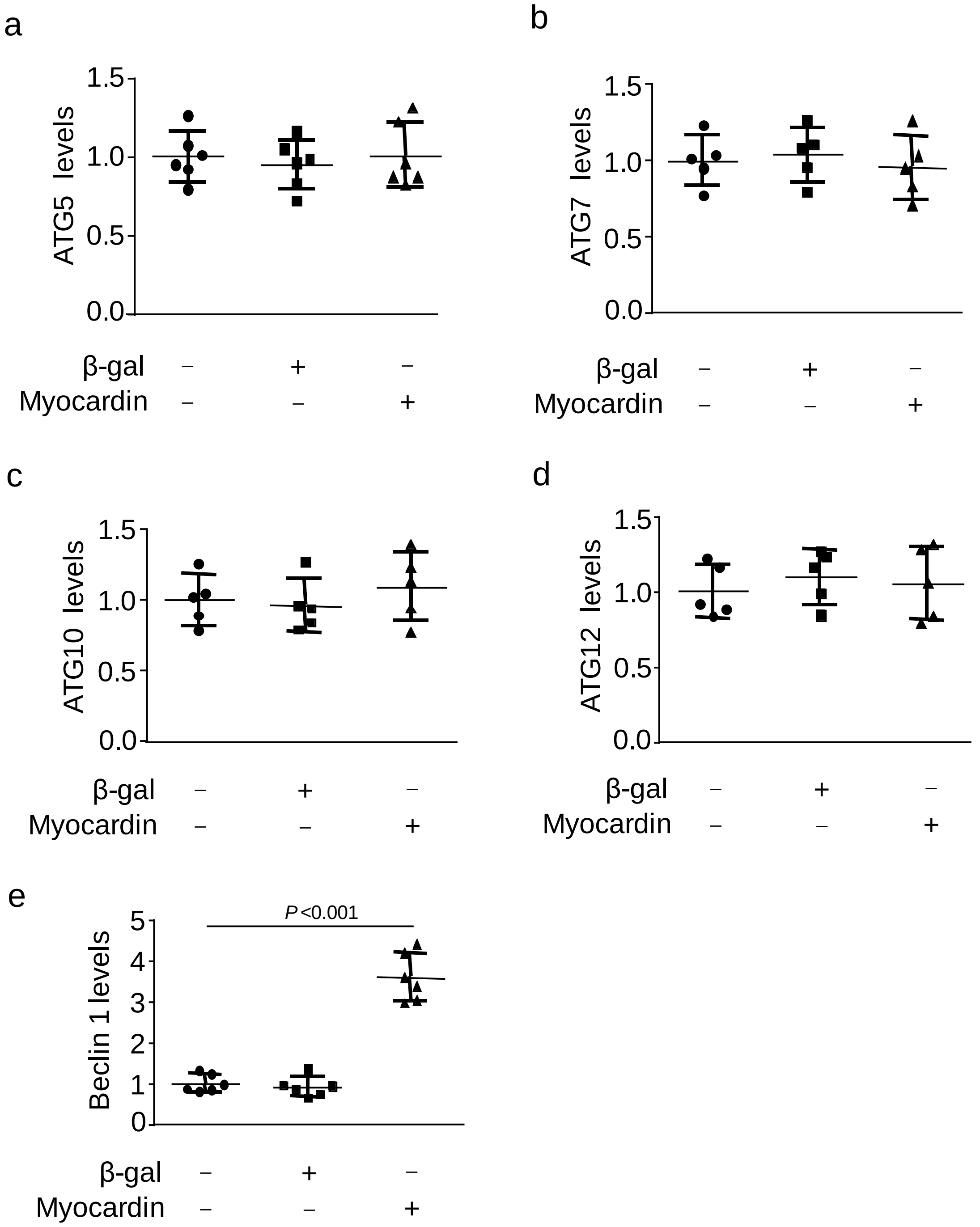

Supplementary Figure 4. Myocardin regulates Beclin 1 expression. (**a-e**) Cardiomyocytes were infected with adenoviral myocardin or β-gal. 48 h after infection ATG5 levels (a), ATG7 levels (b), ATG10 levels (c), ATG12 levels (d) and Beclin 1 levels (e) were analyzed by qRT-PCR. n=5-6.

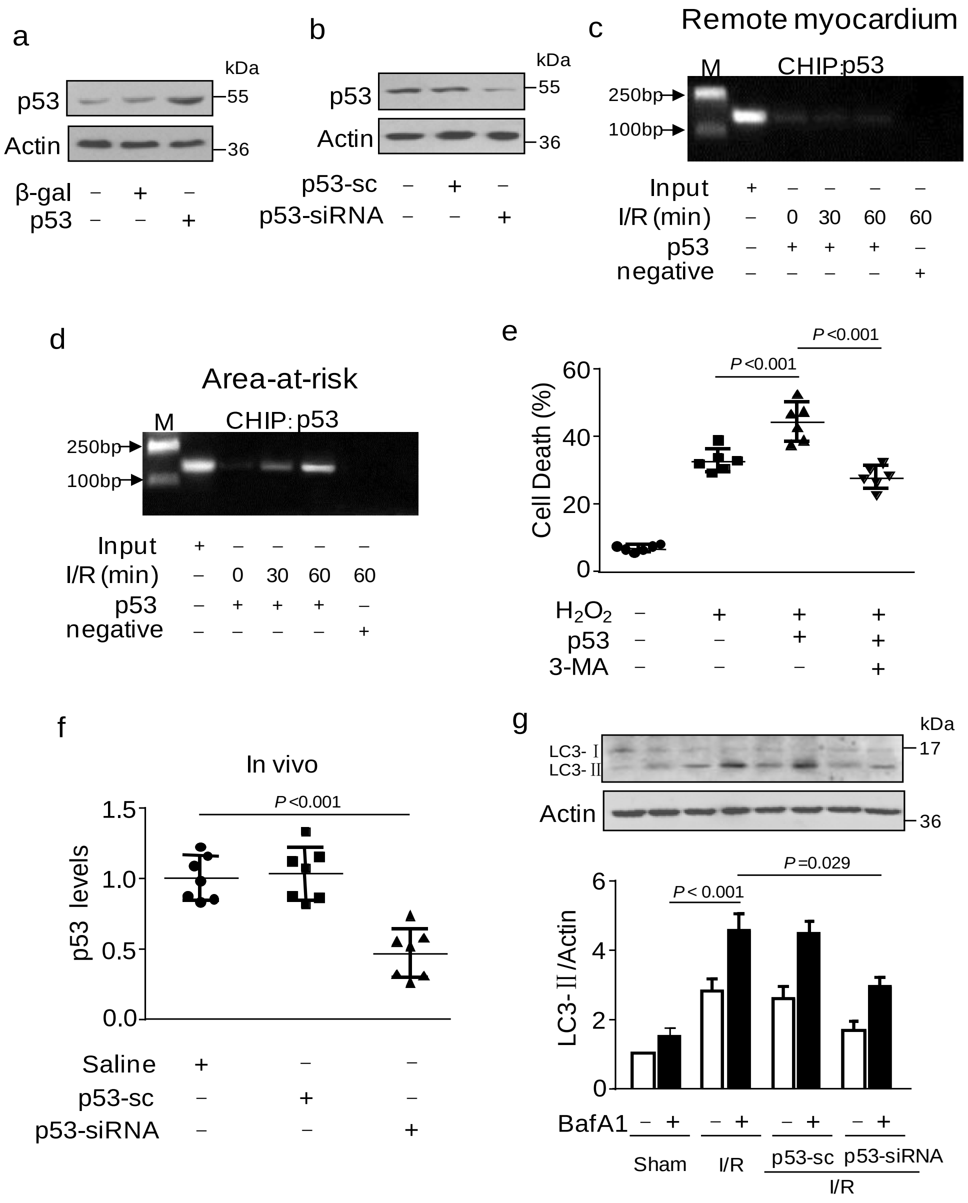

Supplementary Figure 5. p53 binds to the promoter of myocardin in vivo. (**a**) Enforced expression of p53 effectively induces the increase of p53 levels. Cardiomyocytes were infected with adenoviral p53 or β-gal. 24h after infection p53 levels were analyzed by immunoblot. (**b**) Knockdown of p53 reduces the expression levels of p53. Cardiomyocytes were infected with adenoviral p53-siRNA or p53-sc. 24h after infection p53 levels were analyzed by immunoblot. (**c** and **d**) Mice were subjected to ischemia at indicated time and 3h reperfusion (I/R). CHIP assays in remote myocardium (c) and in area-at-risk (d) were performed with p53 or β-actin (negative) antibody. (**e**) p53 induces autophagic cell death. Cardiomyocytes were infected with adenoviral p53, and then treated with H_2O_2 in the presence or absence of 3-MA. 3-MA was preincubated with cells for 1h. Cell death was determined. n=6. (**f**) Mice were injected with adenovirus p53-siRNA or p53-sc as described in methods, p53 levels were analyzed by qRT-PCR. n=7. (**g**) p53 knockdown inhibits cardiac

autophagic flux. Mice were injected with adenoviral p53-siRNA or p53-sc as described in methods, and then were subjected to 45 min ischemia and 3h reperfusion (I/R). BafA1 was administered to mice 2 hours before they were euthanized. Representative western blots showing LC3 expression in the heart samples taken from the area at risk. n=6.

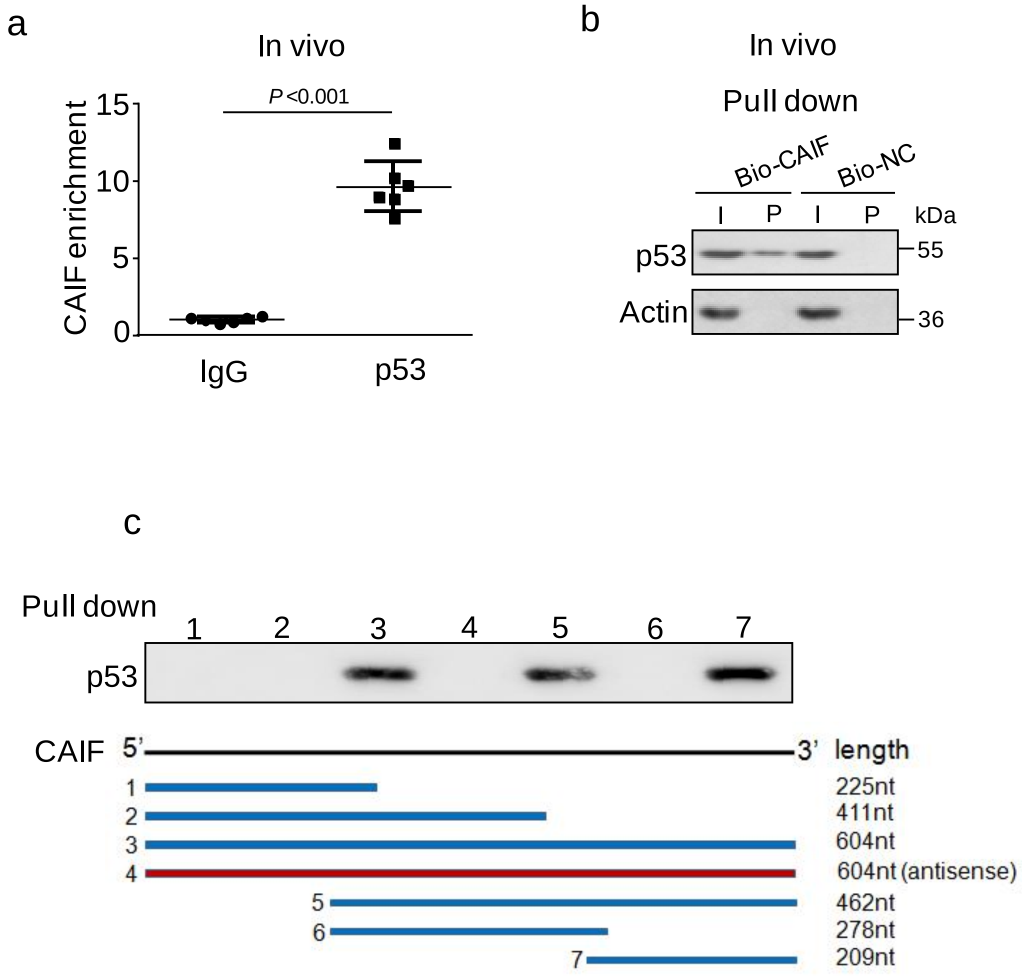

Supplementary Figure 6. CAIF physically interacts with p53. (**a**) RIP assay in vivo. The heart samples from mice were subjected to RIP assay using an anti-p53 antibody or IgG. IP-enriched CAIF was then analyzed by qRT-PCR. n=6. (**b**) RNA pull-down assay was performed in the heart samples from mice using biotin-labeled CAIF probe (Bio-CAIF) and negative control probe (Bio-NC). p53 expression was detected by western blot. (**c**) CAIF binds p53 through its 3' terminal region. RNA pull-down assay was performed in cardiomyocytes using biotin-labeled probes of different RNA fragments of CAIF and associated p53 was detected by western blot.

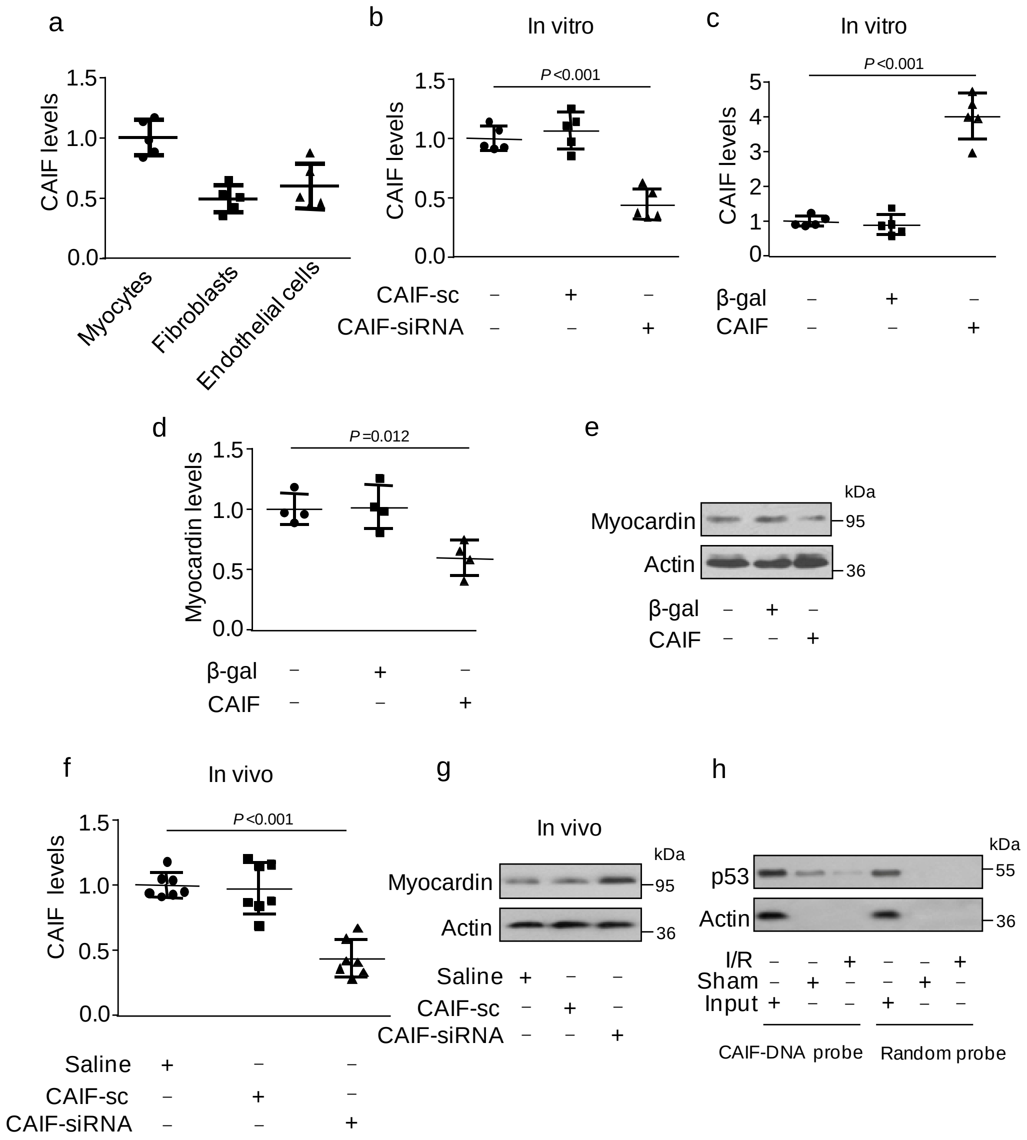

Supplementary Figure 7. CAIF regulates the expression of myocardin. (**a**) The expression levels of CAIF were analyzed by qRT-PCR in different heart cell types (myocytes, fibroblasts and endothelial cells). n=5. (**b**) Knockdown of CAIF decreases CAIF expression. Cardiomyocytes were infected with adenoviral CAIF-siRNA or CAIF-sc. 24h after infection CAIF levels were analyzed by qRT-PCR. n=5. (**c**) Enforced expression of CAIF induces the increase of CAIF levels. Cardiomyocytes were infected with adenoviral CAIF or β-gal. 24h after infection CAIF levels were analyzed by qRT-PCR. n=5. (**d** and **e**) CAIF induces a decrease in myocardin levels. Cardiomyocytes were treated as described in (c). Myocardin mRNA levels were analyzed by qRT-PCR (d). n=4. Myocardin protein levels were analyzed by immunoblot (e). (**f** and **g**) Mice were injected with adenovirus CAIF-siRNA or CAIF-sc as described in methods, CAIF levels were analyzed by qRT-PCR (f). n=7. Myocardin protein levels were analyzed by immunoblot (g). (**h**) I/R decreases the interaction between CAIF and p53 in vivo. Mice were subjected to 45 min ischemia

and 3h reperfusion (I/R). RNA pull-down assay was performed in the heart samples taken from the area at risk using biotinylated DNA probe complementary to CAIF (CAIF-DNA probe) and Random probe. p53 expression was detected by western blot.

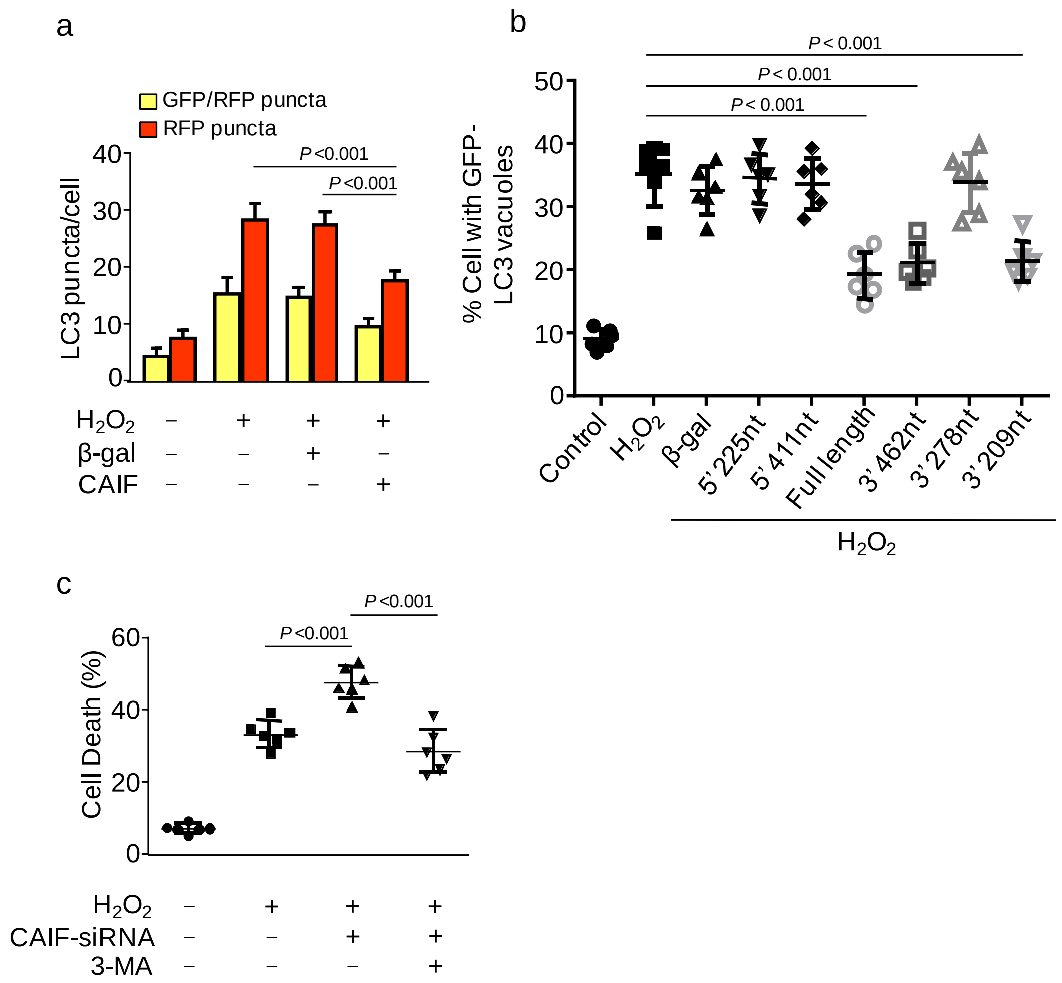

Supplementary Figure 8. CAIF suppresses autophagy. (**a**) CAIF inhibits autophagy flux. Cardiomyocytes were infected with the adenovirus RFP-GFP-LC3 and CAIF or β-gal, 24 h later, treated with H_2O_2 for 24 h. Numbers of autophagosomes (yellow puncta) and autolysosomes (red puncta) in each cell were quantified (n=50 cells per group). n=5. (**b**) Percentage of GFP-LC3 puncta-positive cardiomyocytes. Cardiomyocytes were infected with adenovirus harboring the indicated CAIF fragments. 24h after infection cells were treated with H_2O_2. The percentage of cells with GFP-LC3 puncta was quantified. n=6. (**c**) CAIF mediates autophagic cell death. Cardiomyocytes were infected with adenoviral CAIF-siRNA, and then treated with H_2O_2 in the presence or absence of 3-MA. 3-MA was preincubated with cells for 1h. Cell death was determined. n=6.

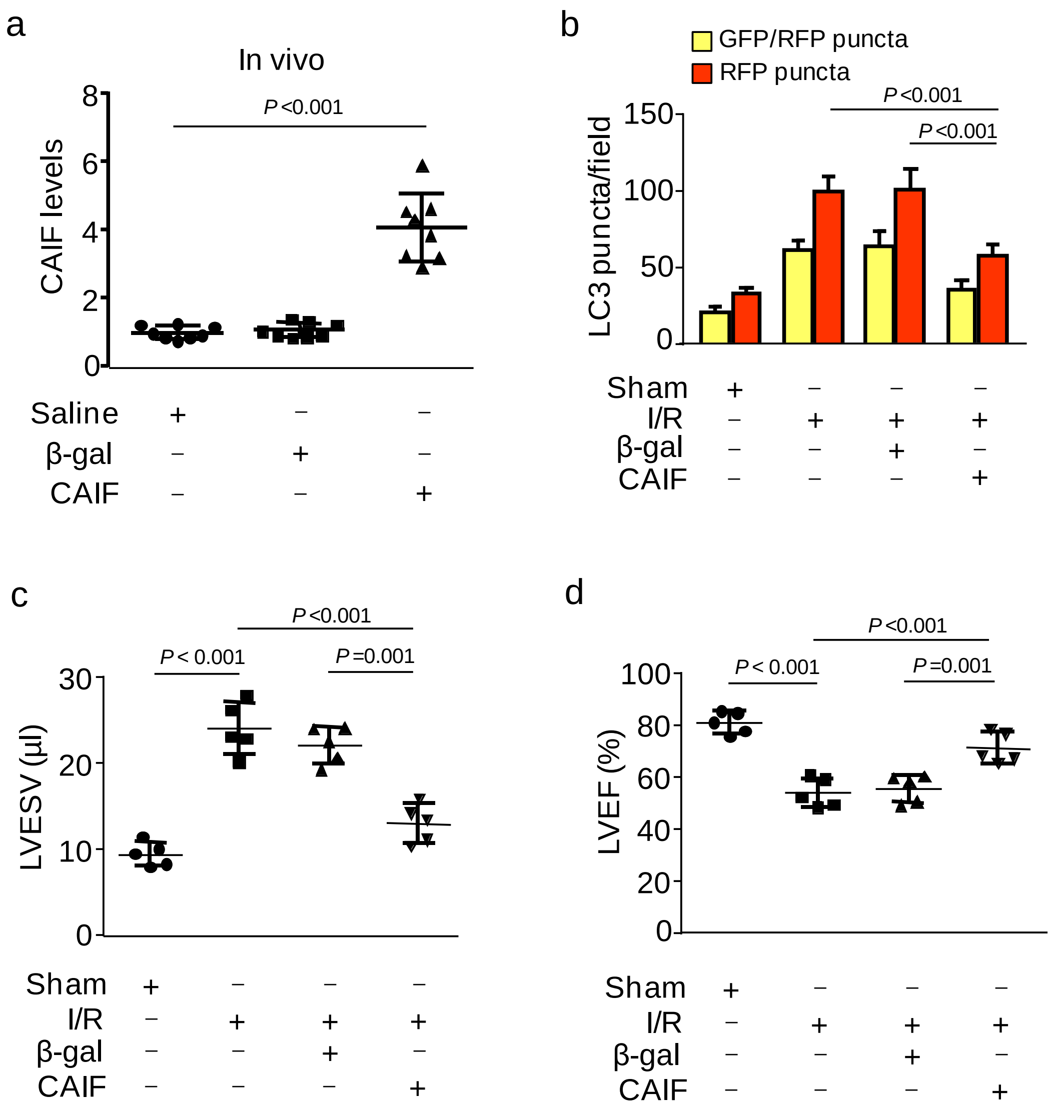

Supplementary Figure 9. CAIF inhibits autophagy and ameliorates cardiac function after I/R. (**a**) Mice were injected with adenovirus CAIF or β-gal and CAIF levels were analyzed by qRT-PCR. n=8. (**b**) CAIF inhibits autophagy flux. Mice were injected with adenovirus RFP-GFP-LC3 and CAIF or β-gal, and then were subjected to 45 min ischemia and 3h reperfusion (I/R). Quantification of GFP and RFP LC3-positive dots per field in the area at risk was shown. n=7 mice per group. (**c** and **d**) Mice were injected with adenovirus CAIF or β-gal as described in methods, then were subjected to 45 min ischemia and 1 week reperfusion (I/R). Echocardiography was employed to test cardiac function. (c) LVESV, left ventricular end-systolic volume; (d) LVEF, left ventricular ejection fraction. n=5.

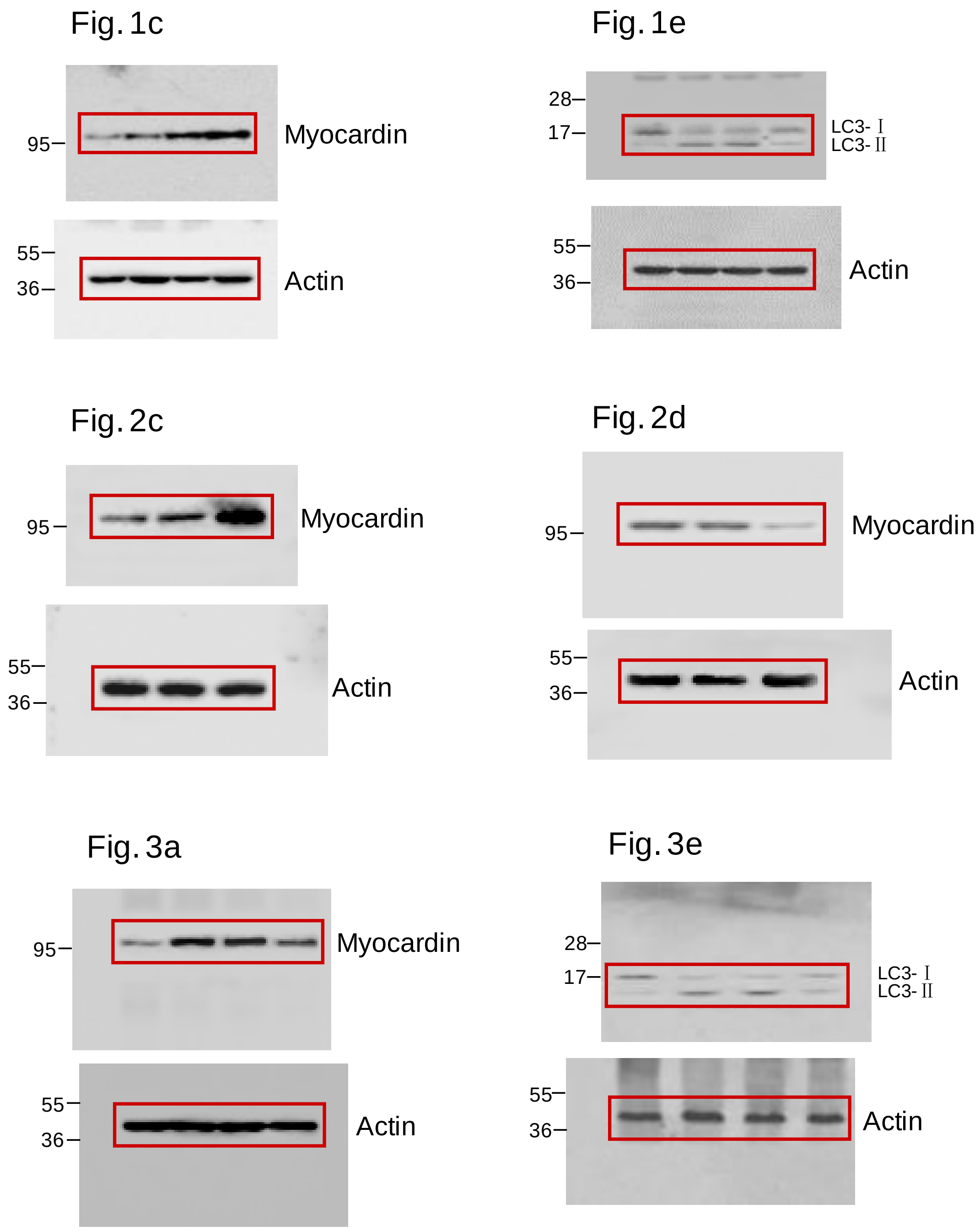

Supplementary Figure 10. Uncropped images of blots presented in the main paper. Molecular weight markers are indicated in kDa.

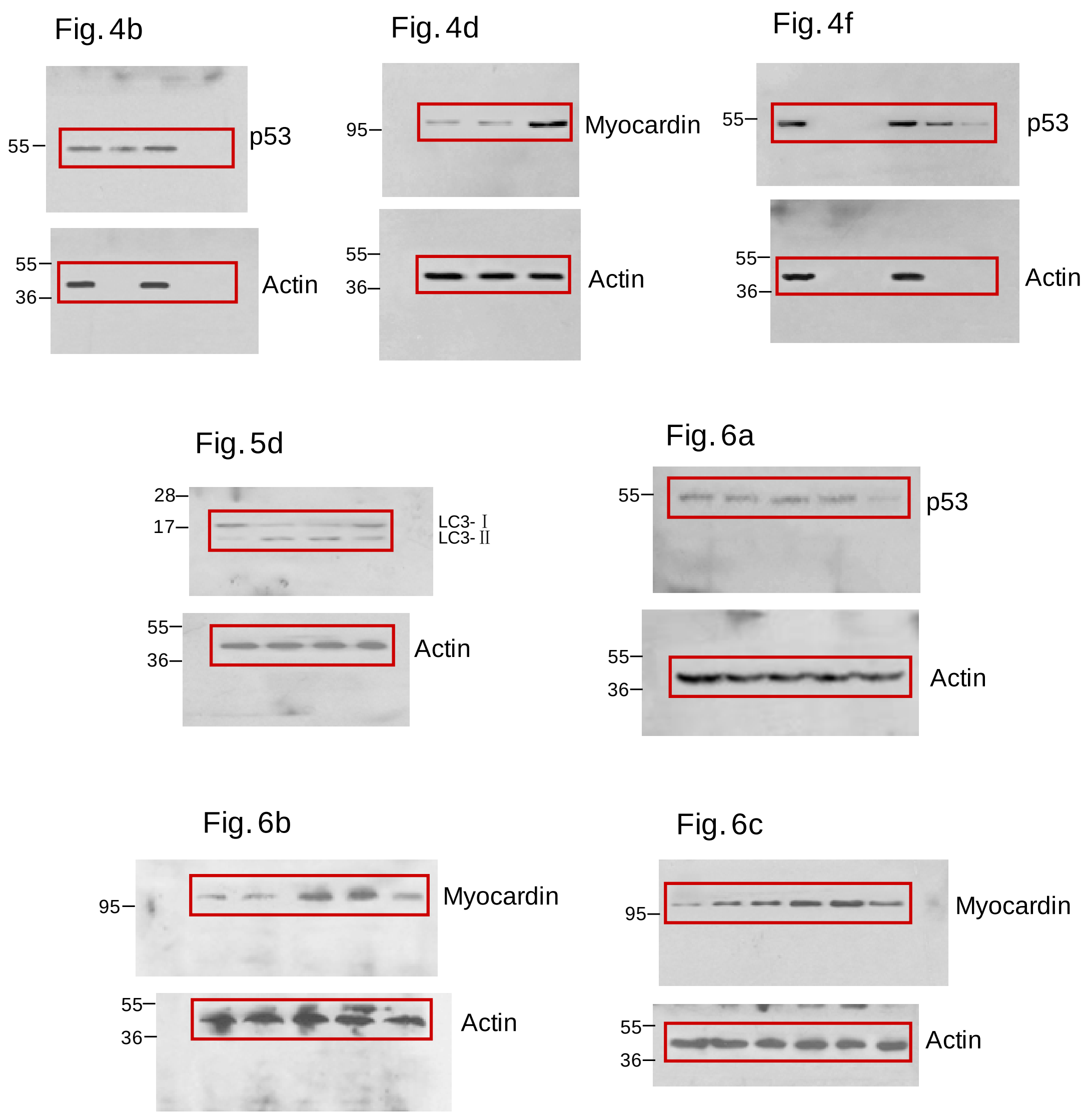

Supplementary Figure 11. Uncropped images of blots presented in the main paper. Molecular weight markers are indicated in kDa.

Supplementary Table 1. LncRNAs expression profile validated via qRT-PCR from control versus H_2O_2 treatment. 50 lncRNAs with high expression levels in heart were chosen from a result of lncRNA array performed by Fantom project.

GenBank Accession numbers	Fold change (H_2O_2/Control)
AK163166	1.076
AK141880	1.120
AK080084	0.893
AK006531	0.786
AK008788	1.074
AK139402	0.831
AK035802	0.979
AK003290	0.473
AK035397	0.962
AK017107	1.133
AK035070	1.067
AK034346	0.872
AK006522	0.948
AK027938	0.964
AK004432	1.247
AK009189	0.904
AK035780	1.350
AK132566	0.929
AK076876	0.532
AK137173	1.073
AK009090	0.917
AK017092	1.163
AK017349	1.085
AK080245	1.124
AK149029	0.609
AK009528	1.236
AK132191	1.144
AK020603	0.872
AK033148	0.967
AK028463	1.047
AK017871	0.983
AK009548	1.141
AK006774	0.495
AK079453	1.314
AK008802	1.267
AK013906	0.874
AK017790	1.059
AK136850	1.218

AK017325	0.785
AK008982	0.921
AK011143	1.134
AK031809	1.279
AK020546	0.426
AK037869	1.278
AK010867	0.894
AK016500	1.225
AK017007	0.917
AK133169	1.225
AK008818	0.807
AK021382	1.148
