# Supplementary material for: LncRNA CAIF inhibits autophagy and attenuates myocardial infarction by blocking p53-mediated myocardin transcription
Source: Nat Commun. 2018 Jan 2;9:29. doi: 10.1038/s41467-017-02280-y (PMC5750208; doi:10.1038/s41467-017-02280-y)
Supplement: Supplementary file 2 — Description of Additional Supplementary Files [file 41467_2017_2280_MOESM2_ESM.pdf]

Description of Additional Supplementary Files

File Name: Supplementary Data 1
Description: Raw data associated with Figure 5G and with Supplementary Figures 1C, 3C, 3D, 3F, 8A, 9B, 9D.
